# Supplementary material for: Nuclear quantum effects on zeolite proton hopping kinetics explored with machine learning potentials and path integral molecular dynamics
Source: Nat Commun. 2023 Feb 23;14:1008. doi: 10.1038/s41467-023-36666-y (PMC9950054; doi:10.1038/s41467-023-36666-y)
Supplement: Supplementary file 1 — Supplementary Information [file 41467_2023_36666_MOESM1_ESM.pdf]

Supplementary Information

Nuclear quantum effects on zeolite proton hopping kinetics explored with machine learning potentials and path integral molecular dynamics

Massimo Bocus‡, Ruben Goeminne‡, Aran Lamaire, Maarten Cools-Ceuppens, Toon Verstraelen and Veronique Van Speybroeck*

Center for Molecular Modeling, Ghent University, Technologiepark 46, 9052, Zwijnaarde, Belgium

*Corresponding author: Veronique.VanSpeybroeck@UGent.be

‡These authors contributed equally.

Contents

Supplementary Note 1 BAS hopping barriers reported in the literature 3

Supplementary Note 2 Static simulations 4

Supplementary Note 3 DFT-US simulation details 5

Supplementary Note 3.1 Extended computational details 5

Supplementary Note 3.1.1 Unit cell equilibration 5

Supplementary Note 3.1.2 US parameters 5

Supplementary Note 3.1.3 FESs computation and error estimation 6

Supplementary Note 3.1.4 Simulation walls 6

Supplementary Note 3.1.5 Plane-wave cutoff convergence 7

Supplementary Note 3.2 FESs 2-D expansion 8

Supplementary Note 3.3 Rate constant calculation 9

Supplementary Note 4 DFT-US results 10

Supplementary Note 5 MLP-US simulation details 14

Supplementary Note 5.1 MLP training 14

Supplementary Note 5.2 Collective variable fine tuning 14

Supplementary Note 6 Classical MLP-US results 15

Supplementary Note 6.1 Free energy profiles 15

Supplementary Note 6.2 Classical rate constants 16

Supplementary Note 7 Path integral MLP-US results 19

Supplementary Note 7.1 Bead convergence 19

Supplementary Note 7.2 Validation against DFT results 19

Supplementary Note 7.3 Free energy profiles 20

Supplementary Note 7.4 Quantum rate constants 24

Supplementary Note 7.5 Quantum rate constants (deuterium) 25

Supplementary Note 8 Final hopping rate calculation 29

Supplementary Note 9 MLP transferability to other zeolite topologies 32

Supplementary Note 10 MLP data efficiency 34

Supplementary Note 1 BAS hopping barriers reported in the literature

Supplementary Table 1: Overview of BAS hopping barriers as derived from the available literature.

Framework	Si/Al	T (K)	E_a ($kJ\cdot mol^{-1}$)	Methodology	Ref.
CHA	11	-	69	Periodic DFT, B3LYP/T(O)DZP, static	[1]
	11	298	73.4	Periodic QM-Pot (+corrections), static	[2]
	11	-	65	Periodic DFT+MP2 corrections (+ZPE), static	[3]
	39	398-548	23±2	IR	[4]
	39	573-773	18±2		
	11	300	71	Periodic DFT, HSE06/PW, blue moon sampling	[5]
	11	600	77		
MFI	21	298-373	11	NMR	[6]
	38	300-660	45	NMR	[7]
	19.5	298-473	17	NMR	[8]
	36	298-473	19		
	53	298-473	20		
	35	370-420	18	NMR	[9]
	15	423-773	89.8	Impedance spectroscopy	[10, 11]
	15	423-773	89.8		
	25	423-773	89.9		
	40	423-773	96.4		
	75	423-773	100.4		
	140	423-773	101.4		
	500	423-773	126.6		
	-	-	117.1	5T cluster, BH&HLYP/6-31G**++ (+ZPE), static	[12]
	95	298	53	Periodic QM-Pot (+corrections), static	[2]
	35	398-548	37±3	IR	[4]
	35	573-773	23±2		
	45	398-548	26±3		
	45	573-773	22±2		
	90	398-548	28±3		
	90	573-773	22±2		
MOR	7	300-660	54	NMR	[7]
	10	398-548	23±2	IR	[4]
	10	573-773	24±2		
FAU	2.6	293-673	31±10	NMR	[13]
	3	300-660	61	NMR	[7]
	2.4	610-640	78	NMR	[9]
	-	-	97.1	3T cluster, MP4/6-311G(d,p) (+ZPE+corrections), static	[14]
	47	298	69.5	Periodic QM-Pot (+corrections), static	[2]

Supplementary Note 2 Static simulations

Preliminary insights in the reactivity differences between the 6 possible hoppings around the Al defect were gained by computing the relative free energy barriers from static simulations. The Vienna Ab Initio Simulation Package (VASP 5.4.4) [15–17] was used, with the Projected Augmented Wave (PAW) method [18, 19]. Energies and forces were computed at a PBE-D3 level of theory [20, 21], with a plane-wave energy cutoff set to 600 eV and a self-consistent field convergence threshold of 10^{-5} eV. The unit cell parameters were fixed at 13.794 Å, 13.784 Å, 14.814 Å, 90.058°, 89.993° and 120.034° for all simulations and the Brillouin zone sampling was restricted to the Γ point.

Transition state structures were initially optimized with the improved dimer method [22] and subsequently refined with a quasi-Newton algorithm [23]. Stationary points were characterized with a Normal Mode Analysis (NMA) in the harmonic approximation, using a Partial Hessian Vibrational Analysis (PHVA) [24] to include all atoms in the first three coordination spheres of the Al defect. This remarkably reduces the computational cost of the simulation while being sufficient for an initial screening of the transition state free energies (as the proton hopping is a very local reaction, we expect the error due to the exclusion of some framework atoms far from the active site from the PHVA to cancel out [25]).

Starting from the relaxed transition state geometries, reactant and products were localized by slightly perturbing the system along the single residual imaginary mode corresponding to the hopping and then fully optimized with the conjugate gradient method. The frequencies of reactants, transition state and products were also used to compute the activation free energy of the hopping at 273 K, 573 K and 873 K, using our in-house developed TAMkin package [26].

The results are summarized in Supplementary Figure 1. The hopping between O_1 and O_4 yields the lowest activation barrier, with a free energy of 53–61 kJ·mol^{-1} (for 273 and 873 K, respectively) using O_3 as reference (which resulted to be the most stable BAS location at all temperatures and, therefore, was chosen as reference). An increase in the activation free energy with temperature can be expected, due to the increasing weight of the entropic penalty in the tensioned transition state. The hopping between O_2 and O_3 also has a low activation barrier, with a free energy of only 54–67 kJ·mol^{-1}. The subsequently lower activated hopping is the one between O_1 and O_2, where the computed barrier is however already 63 kJ·mol^{-1} at 273 K. All the other hoppings have higher activation barriers, with the highest free energy observed for the hopping between O_3 and O_4 (71–86 kJ·mol^{-1}).

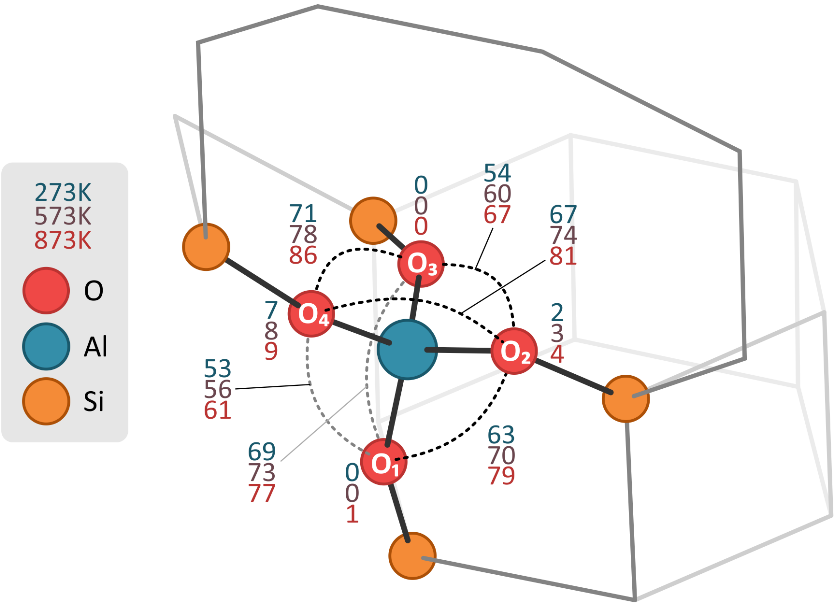

Supplementary Figure 1: Schematic depiction of the CHA framework around the Al defect, where the 6 possible hopping paths are indicated with dotted lines. The numerical values are the transition state free energies or the free energy of the BAS residing on a specific oxygen at 273 K, 573 K and 873 K. All values are referenced to the proton located on O_3, which resulted to be the most stable location at all temperatures.

Concerning the BAS location, there does not seem to be a strongly preferred site around the Al, as they all have free energy differences within 10 kJ·mol^{-1} at all temperatures, in line with previous literature reports [27]. These results suggest that among the various hoppings the O_1–O_4 and O_2–O_3 should be predominant with respect to the others (this will also be confirmed by the dynamic simulations). We therefore decided to use O_2–O_3 as a case study for all the various benchmarks performed within this work.

Supplementary Note 3 DFT-US simulation details

Supplementary Note 3.1 Extended computational details

Supplementary Note 3.1.1 Unit cell equilibration

In order to speed up the simulations and, at the same time, reduce the number of degrees of freedom in the system, we performed all production runs in the NVT ensemble. Considering, however, the large span of temperatures that were investigated, we started by performing three independent 30 ps simulations in the NPT ensemble at 273, 573 and 873 K, using an MTK barostat [28] to apply a pressure of 1 atm. The time evolution of the cell vector lengths as well as the cell volume distributions are shown in Supplementary Figure 2.

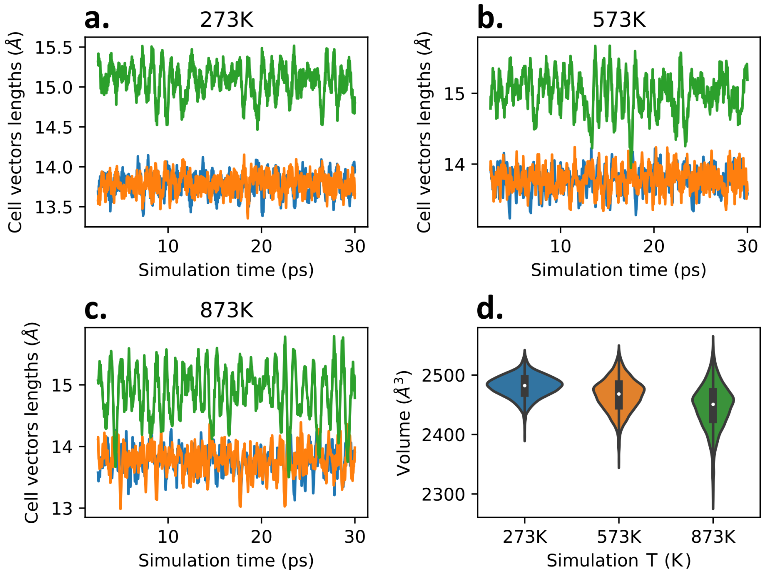

Supplementary Figure 2: Results of the NPT equilibration runs. *a-c.* Time evolution of the unit cell parameters at 273, 573 and 873 K and a pressure of 1 atm. *d.* Violin plot highlighting the shrinking of the average unit cell volume with increasing temperatures.

As it is well-know in literature [29, 30], the CHA topology exhibits a negative thermal expansion coefficient, meaning that the cell volume shrinks with increasing temperature. However, as shown in Supplementary Figure 2, the span of cell vector lengths and volumes explored for the three temperatures mostly overlaps. Moreover, it can be reasonably assumed that very local phenomena, like the proton hopping investigated herein, will hardly depend on small variations in the cell volume. Therefore, we decided to take the average unit cell parameters (13.7733 Å, 13.7824 Å, 14.9983 Å, 90.06°, 90.05°, 119.91°) of the intermediate temperature (573 K) and use these for all the subsequent production runs in the NVT ensemble.

Supplementary Note 3.1.2 US parameters

To uniformly sample the BAS hopping reaction, a set of umbrellas was applied at regular intervals along the collective variable (q) space between reactant and products (see Methods section in the main manuscript) and, subsequently, a DFT MD simulation performed in each of them. Every umbrella is defined as a quadratic potential:

$$V(q) = \frac{1}{2}K(q - q_0)^2, \tag{S3.1}$$

where K is the umbrella's force constant and q_0 its center. Guided by previous works within our group [31], a force constant of 1000 kJ·mol^{-1} was chosen for all umbrellas except for the two located in the minima ($q_0 = -0.9$ and $q_0 = 0.9$) for which, given the expected flatter free energy landscape, a K of 250 was chosen instead. For most of the hoppings, we found that an inter-umbrella spacing of 0.1 allows for a sufficient overlap between umbrellas. For every reaction, the resulting free energy was expanded as a function of the constituent coordination numbers, to ensure that no important regions of the phase space were undersampled (see Supplementary Note 3.2). We found that for the higher-activated hoppings some undersampling was present and, therefore, additional two-dimensional umbrellas were applied (Supplementary Note 4). In such cases, the 2-D umbrellas were centered where the undersampling was present with a κ of 1000–2000 kJ·mol^{-1} in both dimensions. A summary of the overall number of umbrellas used in each hopping simulation is shown in Supplementary Table 2.

Supplementary Table 2: Overview of the number of umbrellas applied in the DFT US simulations for each of the hoppings around the Al defect. For the low-temperature (273 K) simulations of the 2–3 and 1–4 hopping more umbrellas were needed with respect to the higher temperatures (573 K and 873 K) as the reduced thermal energy decreases the collective variable span explored in each simulation.

Hopping	# 1-D umb.	# 2-D umb.
1–2 (873K)	19	3
1–3 (873K)	21	2
3–4 (873K)	19	2
1–4 (873K)	19	–
1–4 (273K)	25	–
2–4 (873K)	19	–
2–3 (873K)	19	–
2–3 (573K)	19	–
2–3 (273K)	25	–

Supplementary Note 3.1.3 FESs computation and error estimation

The time series of the collective variable values explored during the US simulations were combined through the Weighted Histogram Analysis Method (WHAM) as implemented in our in-house developed ThermoLIB library [32]. One step every 5 fs was considered to limit data correlation and retrieve more robust statistics on the free energy surfaces (FESs). Analogously to what has recently been done by some of the authors [31], the standard deviation on the free energy values associated with each bin during the WHAM analysis is derived from the Fisher information matrix which, in its turn, is constructed by interpreting the WHAM equations as a maximum likelihood estimate [33, 34]. In Supplementary Figure 4, Supplementary Figure 5–Supplementary Figure 7, Supplementary Figure 10 and in Figure 2 of the main manuscript, the error bars associated with each point on the FES correspond to the 95% confidence interval, given by twice the standard deviation.

Supplementary Note 3.1.4 Simulation walls

Some of the hoppings proved to be quite difficult to describe because of the highly energetic transition state. In such cases, spontaneous jumps of the proton towards other oxygens in the unit cell were observed. To avoid this, a set of lower quadratic walls was used to prevent these undesired reactions. The wall potential $W(q)$, applied on a certain collective variable q, starts to act when the latter becomes smaller than a certain chosen value q_0, *i.e.*

$$\begin{cases} W(q) = \frac{1}{2}K(q-q_0)^2 & \text{if} \quad q < q_0 \\ W(q) = 0 & \text{if} \quad q \geq q_0 \end{cases} . \tag{S3.2}$$

A full list of the applied walls can be found in Supplementary Table 3. As the walls' purpose is to prevent undesired proton hoppings, the chosen variables q on which the walls act are analogous to the ones used for the investigation of the 'main' hoppings (see Methods section in the main manuscript), where the

system is however not allowed to cross the transition state region. This wall selection, based on trial and error, is far from ideal as quite some restraint is applied to the system and the side reactions are sometimes not fully prevented. By expanding the free energy profiles in two dimensions (see Supplementary Note 3.2), we noticed that all the undesired states are located at $q' = \mathrm{CN}(\mathrm{O}_i; H) + \mathrm{CN}(\mathrm{O}_j; H) < 0.65$ (see Supplementary Figure 5 and Supplementary Figure 6). Therefore, in the subsequent MLP simulations, a single lower wall was placed on such collective variable ($q_0 = 0.65$, $K = 10000$ kJ·mol^{-1}), which proved to be very effective in preventing all undesired side-reactions.

Supplementary Table 3: Complete list of parameters for the walls used in the DFT US simulations of the various hoppings. A schematic overview of the oxygens' nomenclature is reported in Supplementary Figure 3. For all coordination numbers (CNs, see definition in Eq. S5.9) $r_0 = 1.4$ Å and $N = 6$.

Hopping	collective variable	q_0	K (kJ·mol^{-1})
1–2	$\mathrm{CN}(\mathrm{O}_1;\mathrm{H}) - \mathrm{CN}(\mathrm{O}_5;\mathrm{H})$	0.0	2000.0
	$\mathrm{CN}(\mathrm{O}_2;\mathrm{H}) - \mathrm{CN}(\mathrm{O}_5;\mathrm{H})$	0.0	2000.0
	$\mathrm{CN}(\mathrm{O}_1;\mathrm{H}) - \mathrm{CN}(\mathrm{O}_6;\mathrm{H})$	0.0	2000.0
	$\mathrm{CN}(\mathrm{O}_2;\mathrm{H}) - \mathrm{CN}(\mathrm{O}_6;\mathrm{H})$	0.0	2000.0
	$\mathrm{CN}(\mathrm{O}_1;\mathrm{H}) - \mathrm{CN}(\mathrm{O}_4;\mathrm{H})$	0.0	2000.0
1–3	$\mathrm{CN}(\mathrm{O}_1;\mathrm{H}) - \mathrm{CN}(\mathrm{O}_7;\mathrm{H})$	0.0	2000.0
	$\mathrm{CN}(\mathrm{O}_3;\mathrm{H}) - \mathrm{CN}(\mathrm{O}_7;\mathrm{H})$	0.0	2000.0
	$\mathrm{CN}(\mathrm{O}_1;\mathrm{H}) - \mathrm{CN}(\mathrm{O}_4;\mathrm{H})$	0.0	2000.0
	$\mathrm{CN}(\mathrm{O}_3;\mathrm{H}) - \mathrm{CN}(\mathrm{O}_2;\mathrm{H})$	0.0	2000.0
3–4	$\mathrm{CN}(\mathrm{O}_3;\mathrm{H}) - \mathrm{CN}(\mathrm{O}_8;\mathrm{H})$	0.0	2000.0
	$\mathrm{CN}(\mathrm{O}_4;\mathrm{H}) - \mathrm{CN}(\mathrm{O}_8;\mathrm{H})$	0.0	2000.0
	$\mathrm{CN}(\mathrm{O}_3;\mathrm{H}) - \mathrm{CN}(\mathrm{O}_2;\mathrm{H})$	0.0	2000.0
	$\mathrm{CN}(\mathrm{O}_4;\mathrm{H}) - \mathrm{CN}(\mathrm{O}_1;\mathrm{H})$	0.0	2000.0

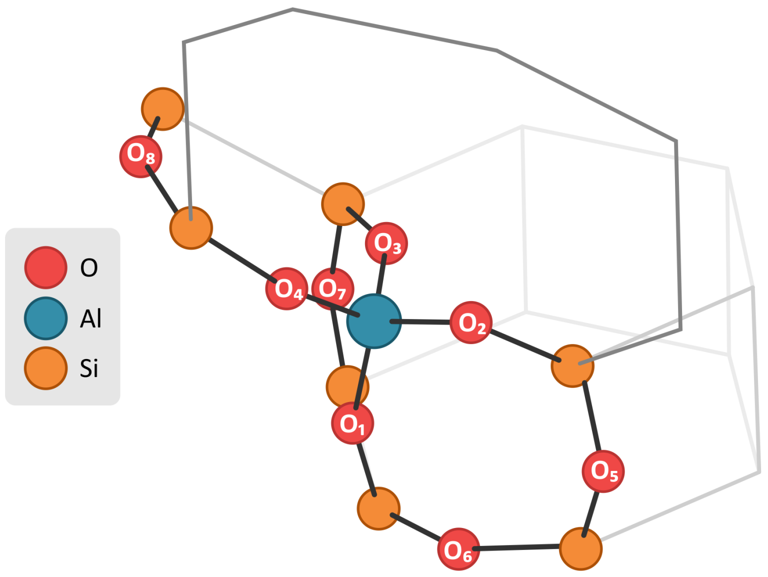

Supplementary Figure 3: Schematic depiction of the H-CHA region around the Al defect, which highlights the conventional nomenclature given to some relevant oxygens (compare with Supplementary Table 3).

Supplementary Note 3.1.5 Plane-wave cutoff convergence

While benchmarking the robustness of the CP2K results with respect to the plane-wave cutoff energy we found that, while energies are already well-converged at a cutoff of 350 Ry, forces can still significantly change when higher cutoffs are used. Therefore, taking the 2–3 hopping at 873 K as case study, we

performed a second DFT US simulation, increasing the plane-wave cutoff from 350 to 800 Ry and the `REL_CUTOFF` from the default 40 Ry to 60 Ry. With these values, the forces are also well-converged. The resulting FES can be almost perfectly superimposed on the FES for the lower cutoffs (Supplementary Figure 4*a*) and the differences in free energy are well within the error bars. Subsequently, 100 random snapshots were extracted from the simulations with 800 Ry cutoff and the forces were recomputed with the lower settings. This was done to compute the Mean Error (ME) on the norm of the difference between the high and low cutoff forces (Eq. S3.3). All oxygen atoms present a relatively large error ($\sim$100 meV·Å^{-1}), while the error for all other atoms is below 1 meV·Å^{-1} (Supplementary Figure 4*b*).

$$\mathrm{ME}_i = \frac{1}{N_{\text{frames}}} \sum_{n=1}^{N_{\text{frames}}} \sqrt{\sum_{c=x,y,z} (f_{i,n,c}^{800\text{Ry}} - f_{i,n,c}^{350\text{Ry}})^2} \tag{S3.3}$$

By comparing the average of the differences between the force components, we also noticed that the error on the oxygen atoms is quite systematic in nature. This different behavior with respect to the other elements could be due to the higher electron density on the oxygen atoms that, being also very diffuse, seem to be more susceptible to a change in plane-wave cutoff with respect to the less electronegative atoms in the system. Despite the error on the oxygen forces, the free energy profile is, as previously stated, basically unaffected and, therefore, we decided to maintain a 350 Ry cutoff with the default `REL_CUTOFF` of 40 Ry, as these settings allow to save a significant amount of computational time.

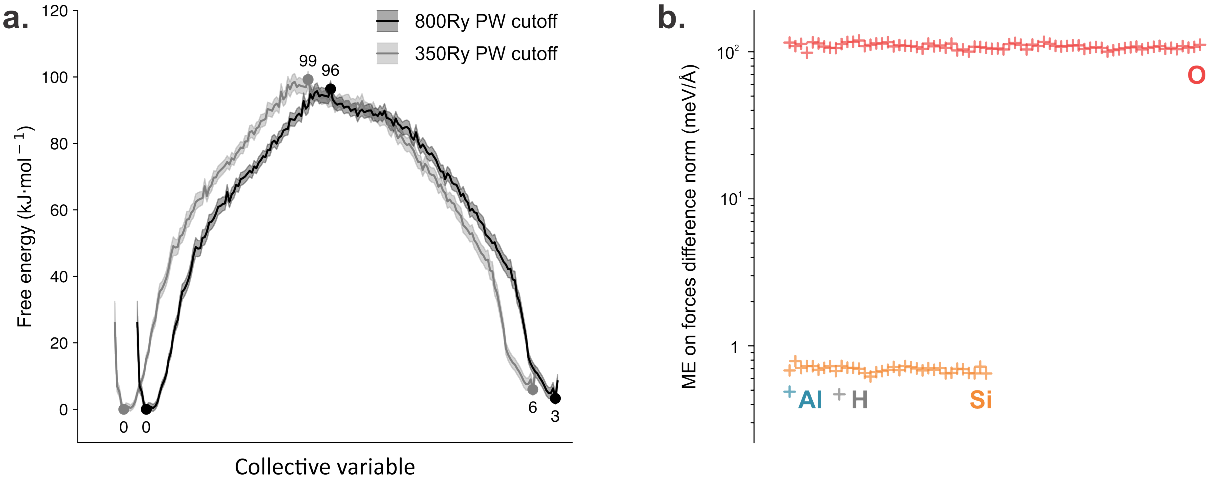

Supplementary Figure 4: Results of the plane-waves cutoff benchmark. *a.* Free energy profiles for the 2–3 hopping at 873 K using a plane-wave cutoff of 350 and 800 Ry. The two profiles have been artificially offset along the collective variable axis to avoid superposition and improve readability. *b.* Mean Error (ME) on the norm of the difference in forces between the high and low PW cutoffs. The oxygen atoms are subjected to an error that is about two orders of magnitude higher than the other atoms in the system.

Supplementary Note 3.2 FESs 2-D expansion

While using a difference in coordination numbers (CNs) as collective variable (q) provides a simple way to reduce the FES dimensionality and perform 1-D US, it can also lead to an undersampling of important regions in the phase space. Using basic statistical analysis, it is possible to expand the 1-D profiles in terms of the constituting coordination numbers, by using the formula:

$$F(q_1, q_2) = -k_b T \ln \left(\int_{-\infty}^{+\infty} p\,(q_1, q_2|q) \exp\left(-\frac{F(q)}{k_b T}\right) \mathrm{d}q \right). \tag{S3.4}$$

This formulation was used to expand the original q in the two CNs that constitute it, *i.e.* $\mathrm{CN}(\mathrm{O}_i; \mathrm{H})$ and $\mathrm{CN}(\mathrm{O}_j; \mathrm{H})$, with i and j being the indices of the two oxygens involved in the considered hopping. While for most of the hoppings a satisfactory coverage of the CN space between reactants and products was found, in some cases (in particular for the most difficult hoppings, with steep free energy barriers to overcome) obvious undersampled regions were present.

To solve this issue and improve the sampling in these difficult regions, we adopted a procedure previously used by some of us in a similar situation [31]. Starting from a two-dimensional space defined by the two original CNs, their difference can also be seen as a 45° rotation of the space together with some stretching. In this new space, the orthogonal direction to the original $q = CN(O_i; H) - CN(O_j; H)$ is given by the sum of the two coordination numbers: $q' = \mathrm{CN}(\mathrm{O}_i; \mathrm{H}) + \mathrm{CN}(\mathrm{O}_j; \mathrm{H})$. Every 1-D umbrella acting on q can, in this way, also be seen as a 2-D umbrella, with a force constant of zero along q'. This allows to easily place additional 2-D umbrellas with non-zero force constants in both directions and combine them with the original ones using a 2-D WHAM analysis, to obtain a final 2-D FES. Thanks to this approach, it was also easy to notice that all states corresponding to unwanted interactions between the proton and other framework oxygens correspond to $q' < 0.6$. All these states were then removed from the 2-D FES before proceeding with the final calculation of the rate constant. Moreover, in the subsequent simulations using the MLP, a single wall was placed on q' to prevent in a clean and controlled manner all unwanted interactions between the BAS and oxygens that are not involved in the hopping of interest.

To retrieve the final reaction rate of the reaction, it is useful to reproject the 2-D FES on the original 1-D collective variable, which can be easily done with the following formula:

$$F(q) = -k_b T \ln \left(\int_{-\infty}^{+\infty} \exp \left(-\frac{F(q, q')}{k_b T} \right) \mathrm{d}q' \right). \tag{S3.5}$$

Supplementary Note 3.3 Rate constant calculation

In the Bennett-Chandler approach to reaction rate calculation, the kinetic constant of a reaction can be written as [35–39]:

$$k_{\mathrm{BC}}(t) = \langle \dot{q}(0)\theta(q(t) - q^*) \rangle_{q(0)=q^*} \frac{e^{-\beta F(q^*)}}{\int_{-\infty}^{q^*} e^{-\beta F(q)} \mathrm{d}q}, \tag{S3.6}$$

where the brackets denote a thermodynamic average (in this case evaluated for trajectories starting on top of the transition state at $t = 0$, *i.e.* $q(0) = q^*$) of the time derivative of q ($\dot{q}$) evaluated at $t = 0$ multiplied by the Heaviside function $\theta(q(t) - q^*)$. The latter gives a contribution of one if at a time t the system has ended up in the product basin and zero otherwise. $F(q)$ denotes the free energy as a function of the reaction collective variable and $\beta = (k_b T)^{-1}$.

Given that the first term of the equation is usually too demanding to compute, since it requires many trajectories to obtain good statistics, one generally resorts to the approximation of transition state theory (TST). In TST, the chances of barrier recrossing are assumed to be zero and an upper limit of the true kinetic constant (*i.e.* the limit for $t \to 0^+$) is computed instead. This leads to the following formulation [40, 41]:

$$k_{\mathrm{TST}} = \lim_{t \to 0^+} k_{\mathrm{BC}}(t) = \langle \dot{q}(0)\theta(\dot{q}(0)) \rangle_{q(0)=q^*} \frac{e^{-\beta F(q^*)}}{\int_{-\infty}^{q^*} e^{-\beta F(q)} \mathrm{d}q} = \sqrt{\frac{1}{2\pi\beta}} \langle |\vec{\nabla}_x q| \rangle_{q=q^*} \frac{e^{-\beta F(q^*)}}{\int_{-\infty}^{q^*} e^{-\beta F(q)} \mathrm{d}q}, \tag{S3.7}$$

where the explicit chance of recrossing is eliminated by using a Heaviside function of the velocity, which can be rewritten as an ensemble average of the q gradient with respect to the mass-weighted coordinates of the system, computed when the system is restrained at the transition state value. While the kinetic constant of a reaction is the macroscopic quantity of interest for an activated process, the values it can assume normally span multiple orders of magnitude, especially if different temperatures are considered. Therefore, for practical purposes, Eyring's equation can be use to convert the kinetic constant in a phenomenological barrier, which allows to perform a more intuitive comparison between the results:

$$\Delta F^{\ddagger}_{\mathrm{phen}} = -\frac{1}{\beta} \ln(k\beta h), \tag{S3.8}$$

with h Plank's constant.

Using the MLP, it was possible to perform multiple independent simulations to obtain an error estimate on the final kinetic constant value, while the error on the DFT TST constants was obtained by considering the prefactor $\langle |\vec{\nabla}_x q| \rangle_{q=q^*}$ and the free energy dependent fraction separately. For the former, an estimate of the uncertainty was obtained with the block averaging method, while for the latter the error bars are

deduced using the method described in Supplementary Note 3.1.3. The final error estimate on $\Delta F^{\ddagger}_{\text{phen}}$ is then obtained through a Monte Carlo procedure, where random value for $\langle|\vec{\nabla}_x q|\rangle_{q=q^*}$ and $F(q)$ are extracted from a normal distribution with a standard deviation computed as previously explained. This is repeated 10 000 times and the final $\Delta F^{\ddagger}_{\text{phen}}$ is given by the average of the results with an uncertainty equal to twice their standard deviation.

Supplementary Note 4 DFT-US results

This section presents a complete overview of the results obtained from the DFT US simulations. For each of the 6 hoppings, we report the one-dimensional FES with the associated uncertainty (see Supplementary Note 3.1.3), its expansion using a second collective variable $q' = \text{CN}(\text{O}_i;\text{H}) + \text{CN}(\text{O}_j;\text{H})$ orthogonal to the original collective variable $q = \text{CN}(\text{O}_i;\text{H}) - \text{CN}(\text{O}_j;\text{H})$ and, finally, a second expansion along the distances H–O_i and H–O_j (more details on the expansion are reported in Supplementary Note 3.2). The latter clearly shows how the easier hoppings are the ones where the BAS is already directed towards the second oxygen in its equilibrium orientation and the minimum is located at distances of 2–3 Å. On the other hand, the highly activated hoppings require a large reorientation of the BAS from its equilibrium orientation, with minima locations that normally exceed 3.5 Å. Unfortunately, in these cases, the larger distances correspond to negligible variations in the CNs constituting the original collective variable and the reorientation process was therefore very difficult to explore, with some distance values showing a clear undersampling. This issue was fixed for the MLP simulations, where the CN parameters were tweaked to smooth the transition between reactants and products, thereby improving the sampling quality (Supplementary Note 5.2).

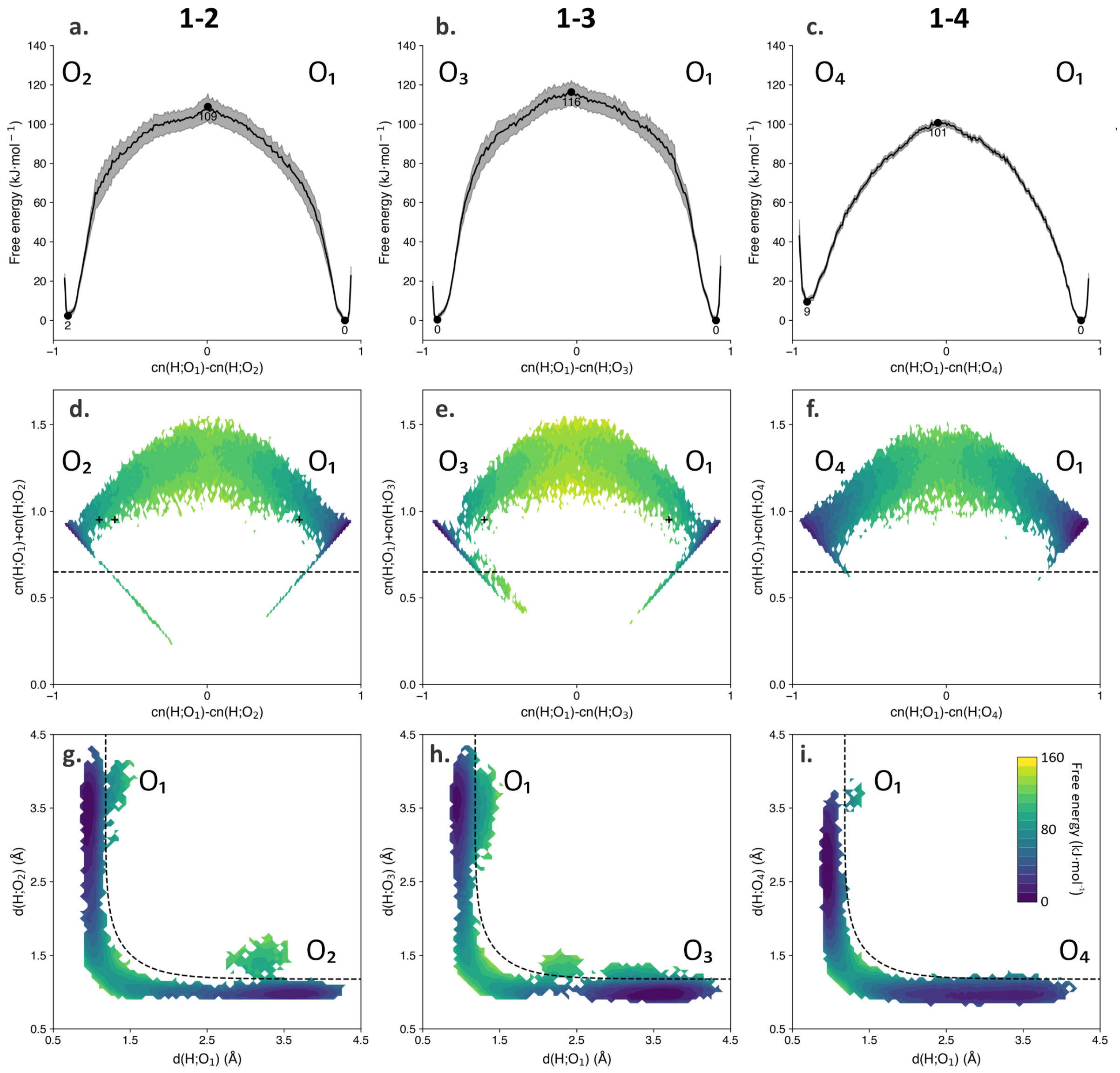

Supplementary Figure 5: 873 K DFT US results. *a–c.* One-dimensional free energy profiles for the 1–2, 1–3 and 1–4 hoppings at 873 K, with the associated uncertainty. *d–f.* Two-dimensional expansion of the free energy profiles as function of the one-dimensional collective variable and its orthogonal direction in the space defined by the two original coordination numbers (see Supplementary Note 3.2). The states below the black dotted line, placed at 0.65, were discarded from the calculation of the rate constant and the phenomenological barrier. Black crosses highlight the center of additional two-dimensional umbrellas. *g–i.* Two-dimensional expansion of the free energy profiles as function of the proton-oxygen distances. The black dotted line corresponds to the one in the surface above, transformed to the new collective variable space.

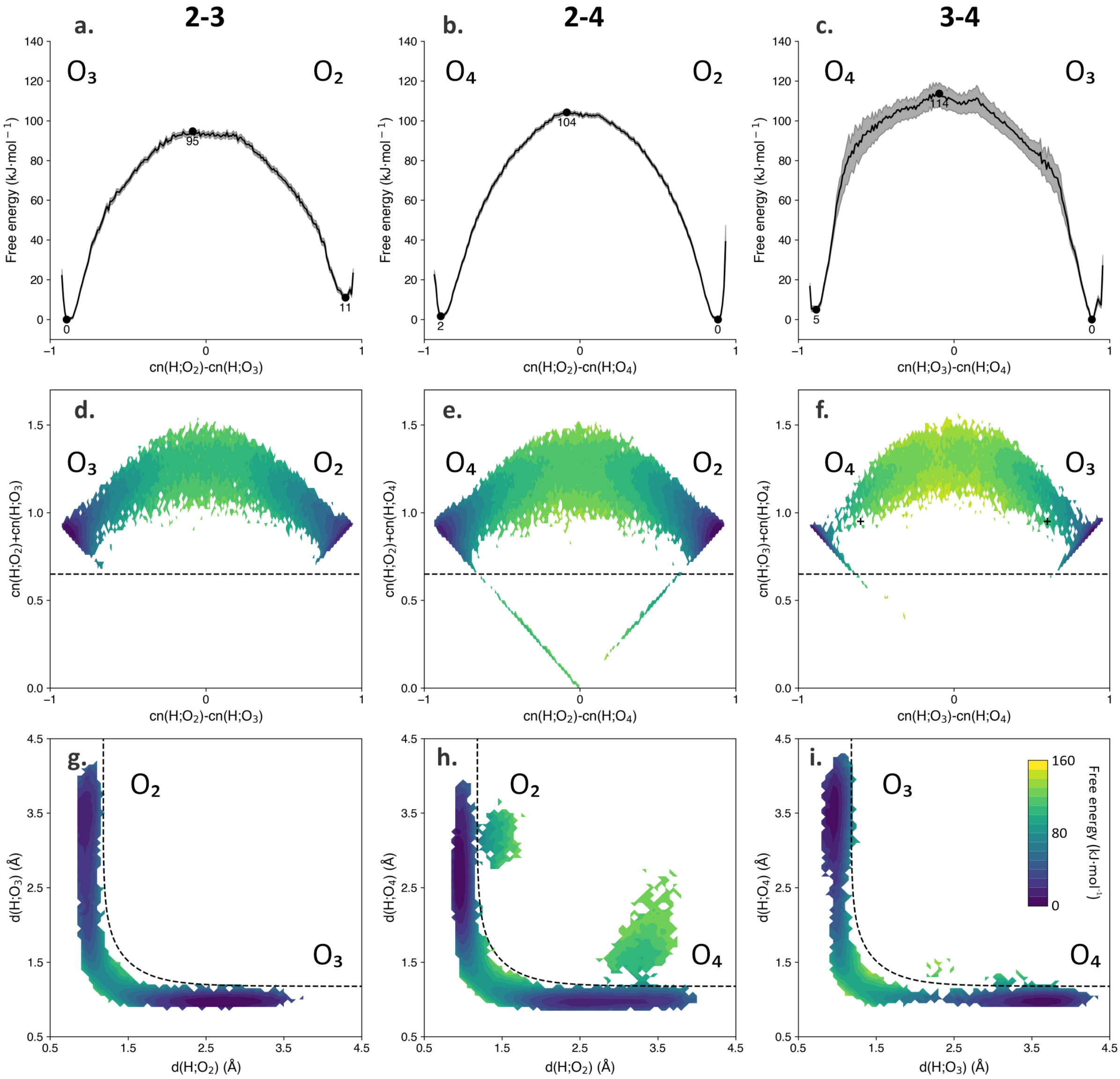

Supplementary Figure 6: 873 K DFT US results. *a–c.* One-dimensional free energy profiles for the 2–3, 2–4 and 3–4 hoppings at 873 K, with the associated uncertainty. *d–f.* Two-dimensional expansion of the free energy profiles as function of the one-dimensional collective variable and its orthogonal direction in the space defined by the two original coordination numbers (see Supplementary Note 3.2). The states below the black dotted line, placed at 0.65, were discarded from the calculation of the rate constant and the phenomenological barrier. Black crosses highlight the center of additional two-dimensional umbrellas. *g–i.* Two-dimensional expansion of the free energy profiles as function of the proton-oxygen distances. The black dotted line corresponds to the one in the surface above, transformed to the new collective variable space.

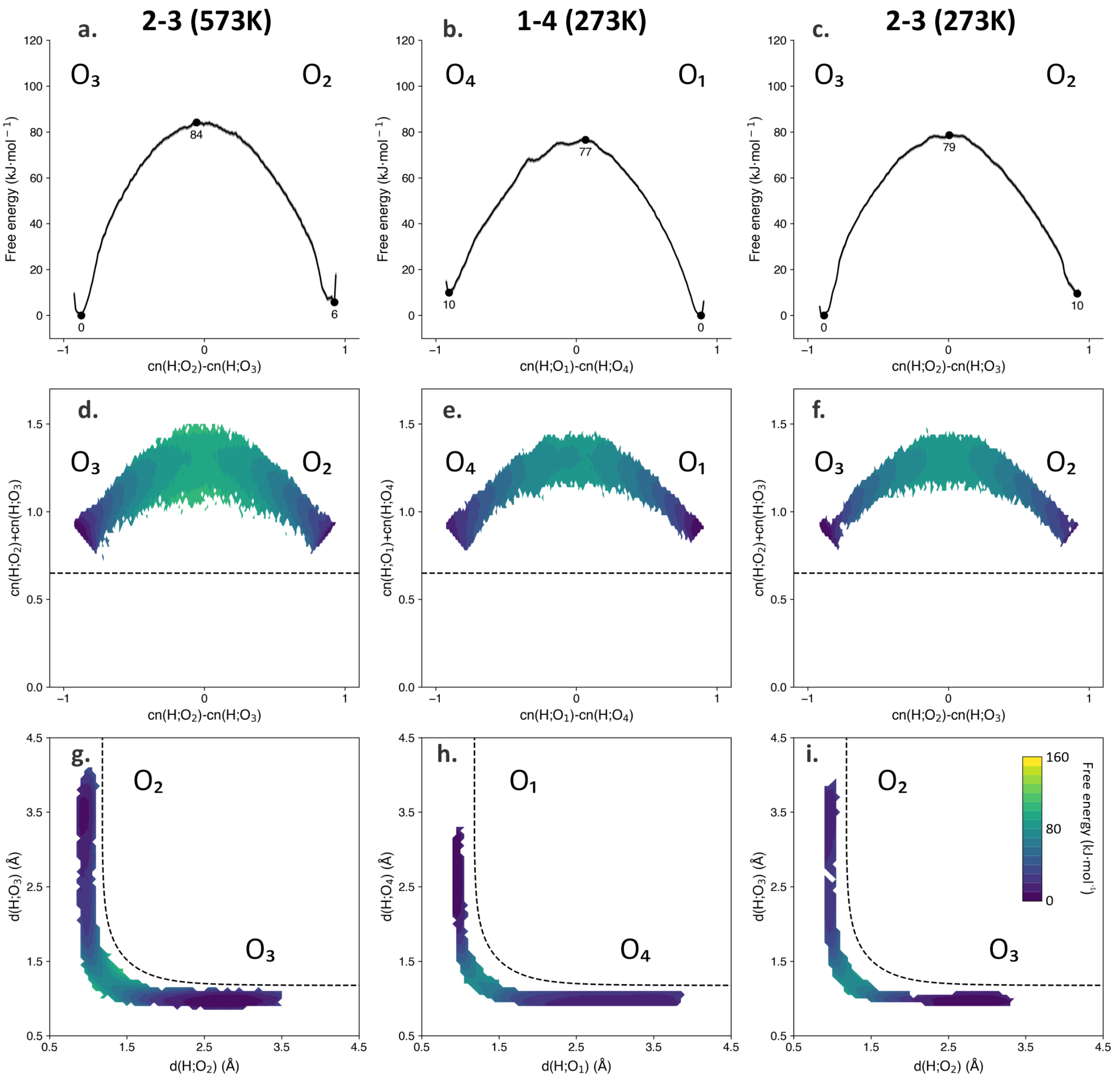

Supplementary Figure 7: 273 and 573 K DFT US results. *a–c.* One-dimensional free energy profiles for the 2–3 hopping at 573 K and the 1–4 and 2–3 hoppings at 273 K, with the associated uncertainty. *d–f.* Two-dimensional expansion of the free energy profiles as function of the one-dimensional collective variable and its orthogonal direction in the space defined by the two original coordination numbers (see Supplementary Note 3.2). The states below the black dotted line, placed at 0.65, were discarded from the calculation of the rate constant and the phenomenological barrier. *g–i.* Two-dimensional expansion of the free energy profiles as function of the proton-oxygen distances. The black dotted line corresponds to the one in the surface above, transformed to the new collective variable space.

Supplementary Note 5 MLP-US simulation details

Supplementary Note 5.1 MLP training

A machine learning potential (MLP) with the SchNet architecture was trained using the open-source `SchNetPack` package. [42, 43] The training data consists of energies and forces that were extracted every 5 fs from the DFT-US simulations of all 6 hoppings at 873 K (see Supplementary Figure 5 and Supplementary Figure 6), resulting in approximately $1.2 \cdot 10^6$ training structures. Before training, the DFT energies and forces were unbiased by subtracting all umbrella and wall biases applied in the US simulations using PLUMED. [44, 45] Subsequently, the SchNet network was trained using a cosine cutoff-function with a cutoff of 6 Å, 128 features, 50 Gaussians and 6 interaction blocks. The model was trained with a batch size of 32 and a learning rate of $2 \cdot 10^{-4}$. In the mean squared error loss function, the energy loss is multiplied with 0.0001 and the force loss with 0.9999. From the data set, 80% is used to train the network, while the remaining 20% is used for validation. The resulting mean absolute error (MAE) on the validation set for the energies and forces is 58.5 meV and 41.9 meV·Å^{-1}, respectively. The validation error during training in shown in Supplementary Figure 8. The observation that the validation error does not noticeably increase further on in training yields a first indication that no overfitting of the model occurs. However, as some correlation still exists between snapshots in the training and validation set, this can only be thoroughly confirmed when performing independent simulations. As will be shown later on (see Supplementary Note 7.2), the MAE on forces of independently performed simulations is 42 meV·Å^{-1}, confirming the lack of overfitting.

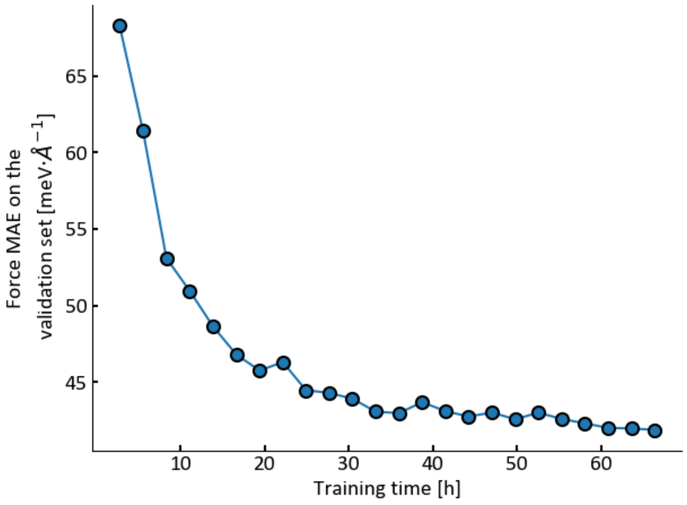

Supplementary Figure 8: Validation MAE on the forces during training of a SchNet model as a function of the training time on 1 NVIDIA V100 GPU. [43]

Supplementary Note 5.2 Collective variable fine tuning

From the expansion of the DFT free energy profiles in the proton-oxygen distances in Supplementary Figure 5 and Supplementary Figure 6, it follows that some under sampling can be present at distances around 2.5 Å from one of the oxygens in the higher-activated hoppings. This is caused by the choice of the parameters N and r_0 in the definition of the collective variable:

$$\mathrm{q} = \mathrm{CN}(\mathrm{O}_i;\mathrm{H}) - \mathrm{CN}(\mathrm{O}_j;\mathrm{H}) = \frac{1-\left(\frac{r_{\mathrm{O}_i\mathrm{H}}}{r_0}\right)^N}{1-\left(\frac{r_{\mathrm{O}_i\mathrm{H}}}{r_0}\right)^{2N}} - \frac{1-\left(\frac{r_{\mathrm{O}_j\mathrm{H}}}{r_0}\right)^N}{1-\left(\frac{r_{\mathrm{O}_j\mathrm{H}}}{r_0}\right)^{2N}}. \quad \text{(S5.9)}$$

As shown in Supplementary Figure 9a, the original choice of the parameters $r_0 = 1.4$ and $N = 6$ results in a collective variable which cannot differentiate states with oxygen-proton distances larger than approximately 2.2 Å (as demonstrated by the lack of variation of the collective variable in this region). Therefore, different values of r_0 (from 1.4 Å to 1.7 Å) and N (from 3 to 6) were scanned for a combination

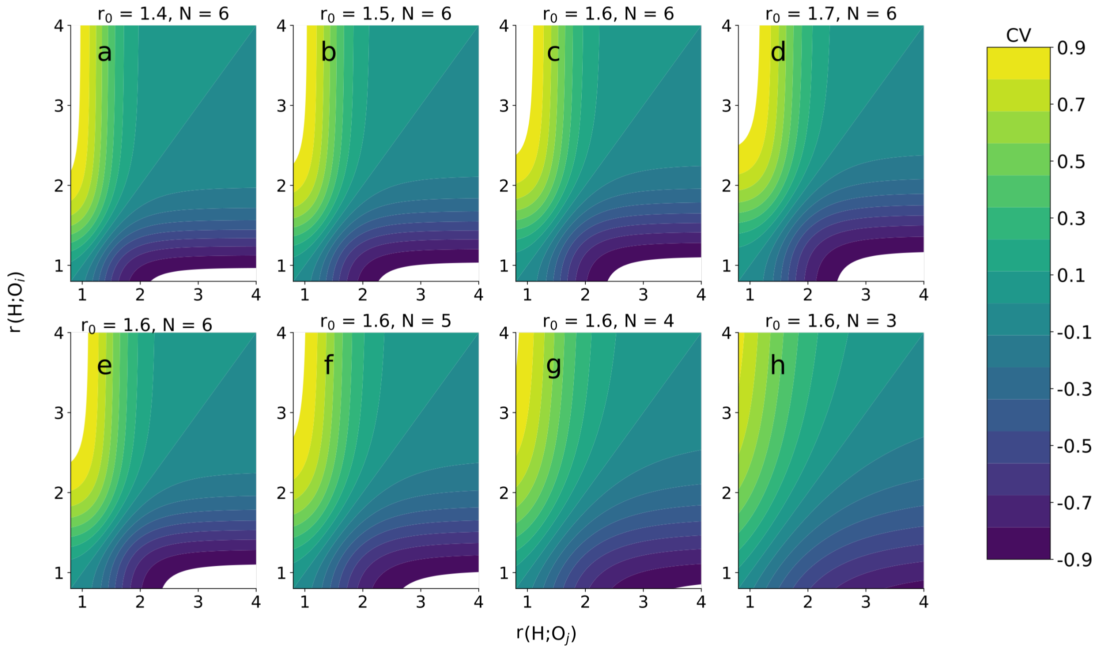

Supplementary Figure 9: Contour plot of the collective variable (Eq. S5.9) as a function of the distance of the proton to both oxygens O_i and O_j for a range of parameters N and r_0. *a-d*: Variation of r_0 for a fixed value of $N = 6$. *e-h*: Variation of N for a fixed value of $r_0 = 1.6$.

of parameters that is able to distinguish these states. A combination of $r_0 = 1.6$ and $N = 4$ was chosen, as this choice allows the collective variable to discriminate states with larger oxygen-proton distances.

The calculations showed that this new choice of collective variable eliminated the under sampling problems for the 1–2, 1–3 and 3–4 hoppings, thereby no longer necessitating the use of two-dimensional umbrellas. For all MLP-US simulations reported in this work, a set of umbrellas centered around values of the new collective variable from -0.95 to 0.95 were applied with a spacing of 0.05 and a force constant K of 1000 kJ·mol^{-1} (see Supplementary Note 3.1.2 for the umbrellas applied in the DFT-US simulations). As mentioned in Supplementary Note 3.1.4, an additional single wall was applied at $q' = \mathrm{CN}(\mathrm{O}_i;\mathrm{H}) + \mathrm{CN}(\mathrm{O}_j;\mathrm{H}) = 0.65$ with a force constant $K = 10000$ kJ·mol^{-1} to prevent undesired side-reactions.

Supplementary Note 6 Classical MLP-US results

Supplementary Note 6.1 Free energy profiles

Supplementary Figure 10 shows a superposition of the classical DFT and MLP free energy profiles for all hoppings at 873 K (except for the 2–3 one, which is reported in Figure 2 of the main manuscript) and the 1–4 hopping at 273 K. The obtained MLP profiles match the DFT profiles nearly perfectly within the uncertainty range. Note that the DFT and MLP simulations were not performed using the same collective variable (Supplementary Note 5.2), but the final FESs can be easily converted to the same collective variable for visualization purposes using the formula (compare with Supplementary Note 3.2):

$$F(q_2) = -k_b T \ln \left(\int_{-\infty}^{+\infty} p\left(q_2|q_1\right) \exp\left(-\frac{F(q_1)}{k_b T}\right) \mathrm{d}q_1 \right). \tag{S6.10}$$

The free energy profiles for each of the 6 hoppings at all the considered temperatures are shown in Supplementary Figure 11. Both the large amount of umbrellas (39 per hopping) and the long simulation times (100 ps) result in well-converged profiles, as seen from the small uncertainty interval from independent simulations.

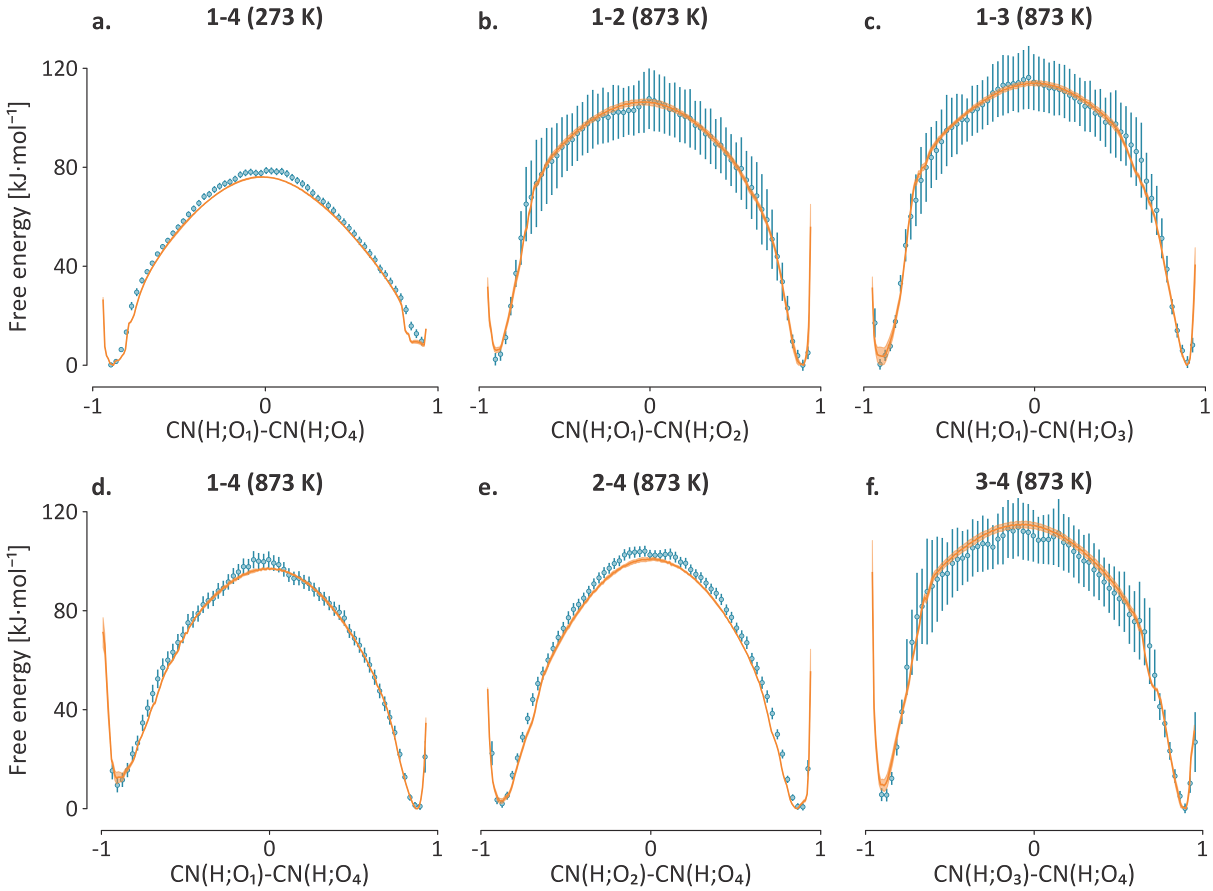

Supplementary Figure 10: Comparison between the DFT-US (blue) and MLP-US (orange) free energy barriers of all hoppings at 873 K (*b–f*, except the 2–3 hopping which is compared in more detail in the main text) and of the 1–4 hopping at 273 K (*a*).

Supplementary Note 6.2 Classical rate constants

As explained in Supplementary Note 3.3, kinetic rate constants are usually computed within the formulation of Transition State Theory (TST), as a direct calculation of the rate constant in the Bennett-Chandler formulation requires additional calculations besides the free energy profile. However, due to the reduced computational cost of the MLP with respect to the DFT simulations, the full time-dependent rate including barrier recrossing can be calculated. To calculate the rate factor (*i.e.* the prefactor in the rate constant)

$$A(t) = \langle \dot{q}(0)\, \theta(q(t) - q^*) \rangle_{q(0)=q^*} \tag{S6.11}$$

5000 configurations around the transition state ($q^* - 0.05 < q(0) < q^* + 0.05$) were extracted from the US simulation for each of the 6 hoppings and 7 temperatures (from 273 K to 873 K). An unbiased NVT simulation, initialized with random velocities sampled from a Maxwell-Boltzmann distribution at the temperature of interest, was then run for 50 fs. In principle, the actual kinetic constant of the reaction is obtained only at the limit for $t \to +\infty$. However, we noticed that – in practice – all trajectories had ended up either in the product or reactant state after 50 fs, without a further possibility to recross the barrier. As shown in Supplementary Figure 12a, the k_{BC} is indeed well converged at the 50 fs mark. In the figure, the ratio between the time-dependent rates and the TST rates for each of the 6 hoppings at 273 K is shown:

$$\kappa(t) = \frac{\langle \dot{q}(0)\, \theta(q(t) - q^*) \rangle_{q(0)=q^*}}{\langle \dot{q}(0)\, \theta(\dot{q}(0)) \rangle_{q(0)=q^*}}. \tag{S6.12}$$

The ratios computed from product to reactant state are observed to be indistinguishable from the ratios computed from reactant to product state. Starting at a ratio of 1 at $t = 0$, $\kappa(t)$ decreases with time due to barrier recrossing and converges after approximately 35 fs in the classical case and 45 fs when

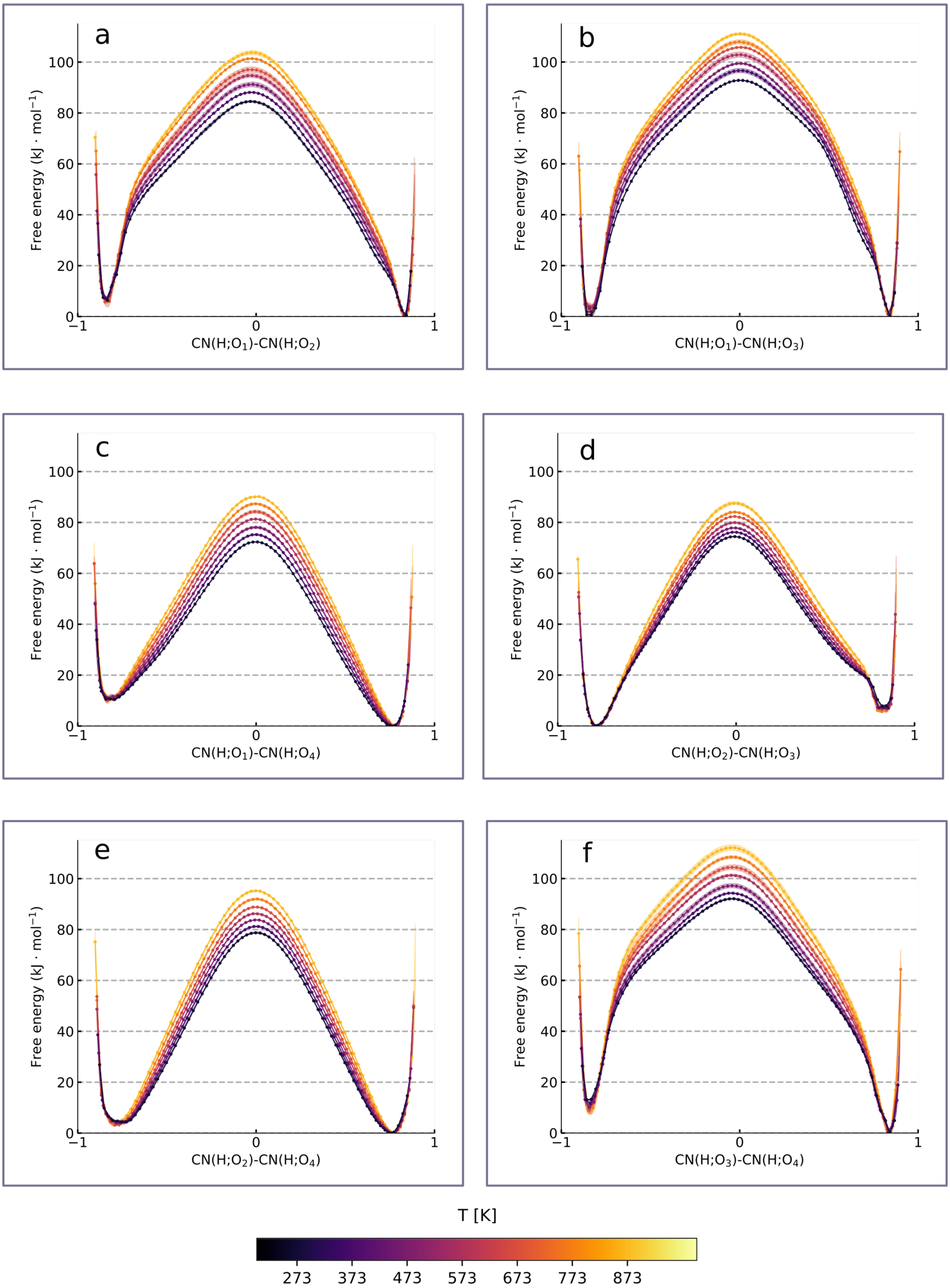

Supplementary Figure 11: MLP-US free energy profiles at temperatures ranging from 273 K to 873 K with a step size of 100 K. *a.* 1–2 hopping. *b.* 1–3 hopping. *c.* 1–4 hopping. *d.* 2–3 hopping. *e.* 2–4 hopping. *f.* 3–4 hopping. Results were averaged over 3 independent simulations, with the shaded area showing the standard deviation between these runs.

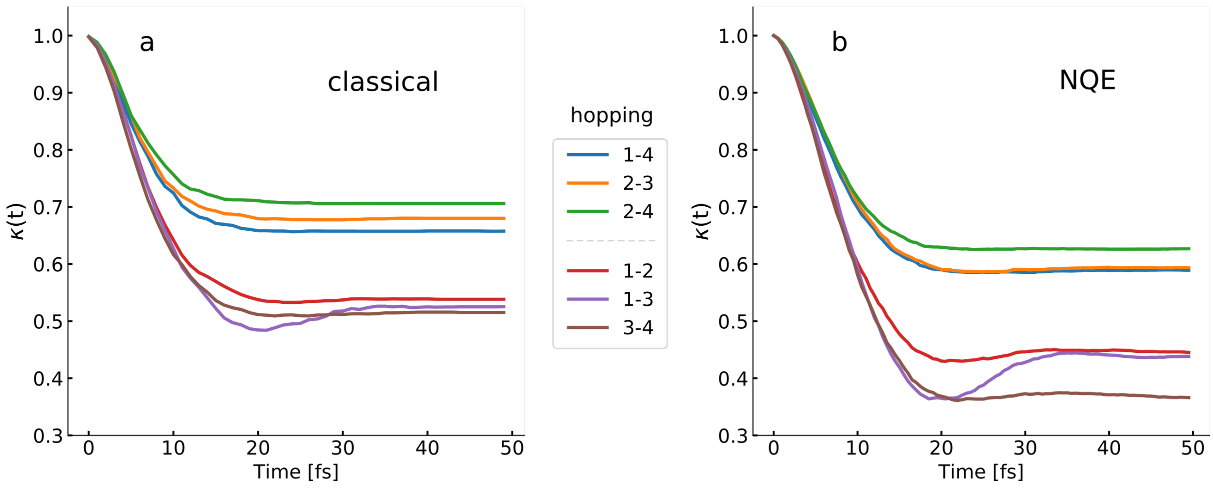

Supplementary Figure 12: Ratio of the time-dependent and TST rate factors (Eq. S6.12) as a function of time at 273 K averaged over 5000 trajectories. *a.* In the classical case. *b.* including nuclear quantum effects. The ratio computed from product to reactant state is observed to be indistinguishable from the ratio computed from reactant to product state.

including nuclear quantum effects (NQEs) (Supplementary Figure 12b). Therefore, the chosen simulation length of 50 fs is sufficient to obtain a converged k_{BC} values. The results for the full time-dependent Bennett-Chandler (BC) and TST kinetic constants are shown in Supplementary Note 7.4 and compared with the BC rates calculated with NQEs.

Supplementary Note 7 Path integral MLP-US results

To perform biased path integral MD (PIMD) simulations, the `i-PI` driver was used to integrate the equations of motion of a ring polymer [46]. Temperature control was achieved with a PILE thermostat [47], which couples a local Langevin thermostat to the centroid of the ring polymers. A time step of 0.25 fs and a simulation length of 25 ps per umbrella was used. Similarly to the classical results, the free energies were averaged over 3 independent simulations.

Supplementary Note 7.1 Bead convergence

In PIMD simulations, the quantum nature of the nuclei is effectively taken into account by means of the classical isomorphism, which replaces every quantum particle by a ring polymer of P harmonically coupled beads [48]. In the limit of $P \to \infty$ this isomorphism yields exact results, but for practical applications one is of course limited to a finite number of beads. To determine an appropriate value for P, the free energy profiles of the 2–3 hopping were calculated at 273 K for different values of P, ranging from 4 to 64. The resulting bead free energy profiles are shown in Supplementary Figure 13. As the difference between the profile obtained using 16 beads and 64 beads is less than 1 kJ·mol^{-1}, all PIMD simulations are performed using 16 beads.

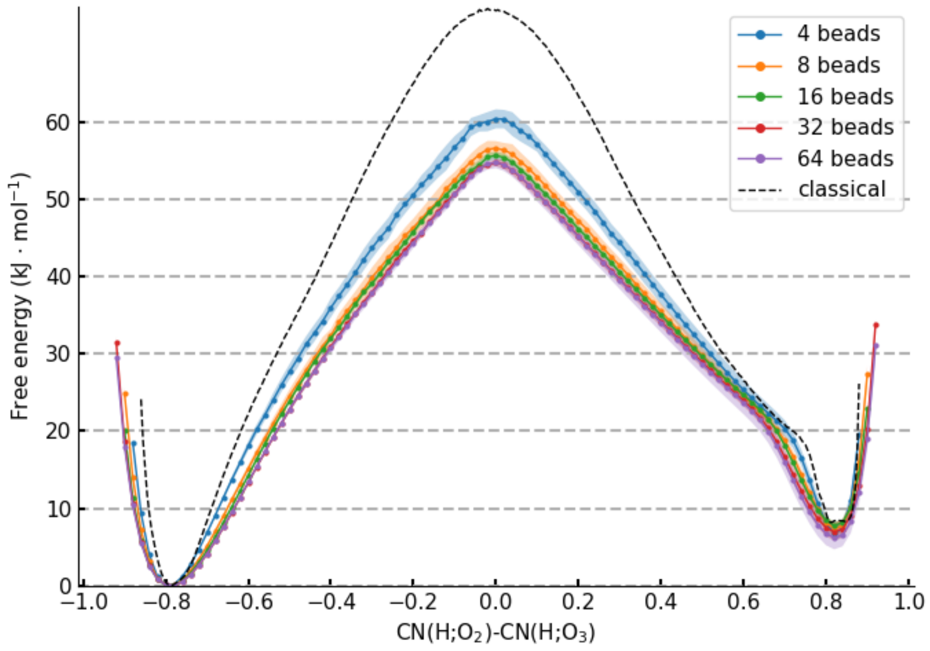

Supplementary Figure 13: Bead free energy profiles of the 2–3 hopping at 273 K calculated classically (1 bead) and using 4, 8, 16, 32 and 64 beads.

Supplementary Note 7.2 Validation against DFT results

Although every bead in a PIMD simulation explores the same potential energy surface as in a classical simulation, we nevertheless validate the PIMD MLP results vs. the DFT ones, as the ring polymer might push some of the beads into regions orthogonal to the optimal reaction path, which have been poorly sampled in the classical simulations. To this end, the 2–3 hopping at 273 K was chosen as case study. Since performing a full US simulation with 16 beads per umbrella would require a prohibitively large amount of computational resources, the MLP was used to construct an initial FES using a metadynamics simulation [49, 50], so that the inverse of the obtained FES could be used as bias for the following DFT-US simulations. This allowed us to reduce the number of umbrellas to only 6, with low force constants ($\sim$25 kJ·mol^{-1}).

Despite the good overall agreement between the MLP and DFT PIMD profiles, with variations in the order of 5 kJ·mol^{-1} (Supplementary Figure 14a), quite some difference can be seen in the steepest

regions of the FES (for a collective variable ≈ ±0.7). This is most likely due to the behavior of the DFT umbrellas located in these regions, where clear non-ergodic behavior in the collective variable time series is present (Supplementary Figure 14b). The MLP simulations do not suffer from this problem, as about 6 times more umbrellas are used for a longer simulation time and with a tighter force constant. To support this argument, we extracted 480 000 frames from the DFT PIMD simulation and computed the MLP MAE on the atomic forces. The results are shown as function of the reaction collective variable in Supplementary Figure 14c. As expected, the MAE on the forces acting on the proton shows some variability with respect to the collective variable, but it never exceeds 50 meV·Å^{-1}. Moreover, no obvious trend is present that might indicate specific issues or inaccuracies in the ±0.7 q region. The obtained MAE on the forces averaged over all atoms of 42 meV·Å^{-1} is the same as the validation error of the MLP (see Supplementary Note 5.1), further demonstrating that no significant overfitting occurred during training.

Given that increasing the number of DFT umbrellas or the simulation time is currently at the edge of our available computational resources, we consider the small differences between the two FESs acceptable given the short time of the DFT simulations and the low error on the forces. Furthermore, this also explicitly stresses how the use of an MLP is pivotal to routinely include NQEs in solid state catalytic reactions.

Supplementary Note 7.3 Free energy profiles

In PIMD, thermodynamic quantities are determined by averaging over the beads of the ring polymer. As all beads are in principle equivalent, this provides a simple way to improve the statistics of ensemble averages. Hence, to calculate the quantum free energy, one needs to compute [51]

$$F(q) = -\frac{1}{\beta} \ln \left\langle \frac{1}{P} \sum_{k=1}^{P} \delta(Q(\mathbf{r}^{(k)^N}, \mathbf{p}^{(k)^N}) - q) \right\rangle = -\frac{1}{P\beta} \sum_{k=1}^{P} \ln \left\langle \delta(Q(\mathbf{r}^{(k)^N}, \mathbf{p}^{(k)^N}) - q) \right\rangle, \tag{S7.13}$$

with $\mathbf{r}^{(k)}$ and $\mathbf{p}^{(k)}$ respectively the position and momentum of bead k, $Q(\mathbf{r}^{(k)^N}, \mathbf{p}^{(k)^N})$ the function representing the collective variable and $\langle \cdot \rangle$ an ensemble average involving the effective PIMD Hamiltonian

$$H_P = \sum_{k=1}^{P} \left[\sum_{i=1}^{N} \frac{\mathbf{p}_i^{(k)^2}}{2m_i} + \sum_{i=1}^{N} \frac{1}{2} m_i \omega_P^2 \left(\mathbf{r}_i^{(k+1)} - \mathbf{r}_i^{(k)} \right)^2 + \frac{1}{P} V(\mathbf{r}_1^{(k)}, \dots, \mathbf{r}_N^{(k)}) \right], \tag{S7.14}$$

with $\omega_P = \frac{\sqrt{P}}{\beta\hbar}$. However, to avoid biasing every single bead (as implied by Eq. S7.13) and the potential sampling difficulties around the transition state that are associated with it (cf. Supplementary Note 7.4), we perform the PIMD enhanced sampling in a pseudo-classical way by biasing the centroid of the ring polymer, defined as

$$\mathbf{r}^{(c)} = \frac{1}{P} \sum_{i=1}^{P} \mathbf{r}^{(i)}. \tag{S7.15}$$

Afterwards, the FES as a function of the centroid collective variable $q^{(c)}$ can be transformed into the quantum FES as a function of the bead collective variable $q^{(k)}$ by a change of variable:

$$F_{\text{quantum}}(q) = -\frac{1}{\beta} \ln \left[\frac{1}{P} \sum_{k=1}^{P} \int \mathrm{d}q^{(c)}\, p(q^{(k)}|q^{(c)})\, e^{-\beta F_{\text{centroid}}(q^{(c)})} \right] + C, \tag{S7.16}$$

with C a constant. To perform this transformation, one only needs to keep track of the values of $q^{(c)}$ and $q^{(k)}$ during the US PIMD simulations. For all 6 hoppings, the quantum free energy profiles were calculated at all 7 temperatures. The results for the 1–4, 2–3 and 2–4 hoppings are shown in Supplementary Figure 15, whereas the results for 1–2, 1–3 and 3–4 hoppings, which exhibit larger barriers, are reported in Supplementary Figure 16.

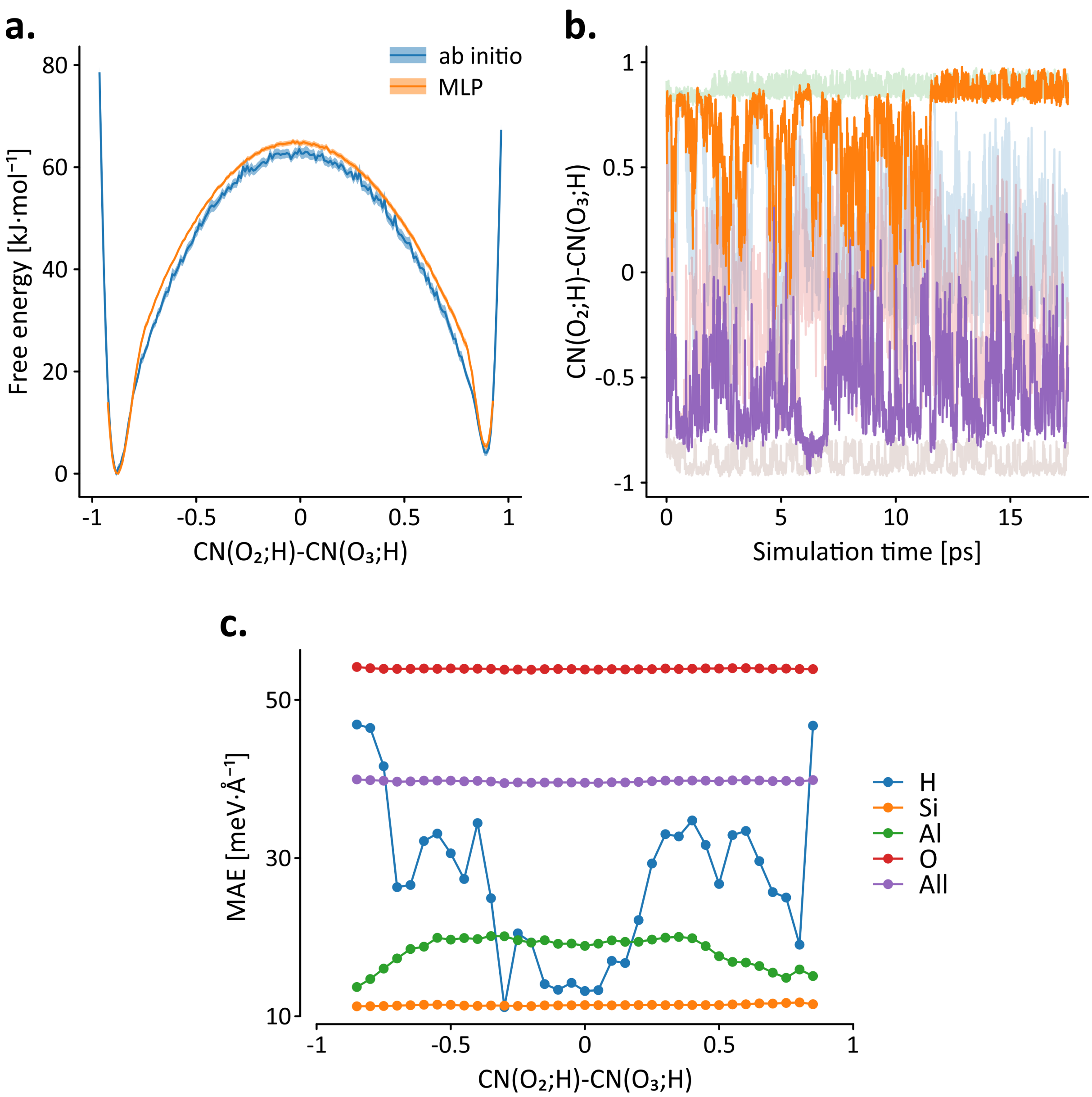

Supplementary Figure 14: DFT validation of the PIMD MLP results. *a.* Centroid free energy profile for the 2–3 hopping as obtained from MLP and DFT US simulations with PIMD. Note that the former is obtained from 39 umbrellas while the latter is obtained from 6 umbrellas (with an additional bias from a previous MLP metadynamics simulation, see Supplementary Note 7.2). *b.* Collective variable time series for the 6 DFT umbrellas. The two umbrellas presenting clear non-ergodic behavior are highlighted with darker colors. *c.* MAE per species on the forces as function of the reaction collective variable, based on structures extracted from the DFT PIMD US simulations.

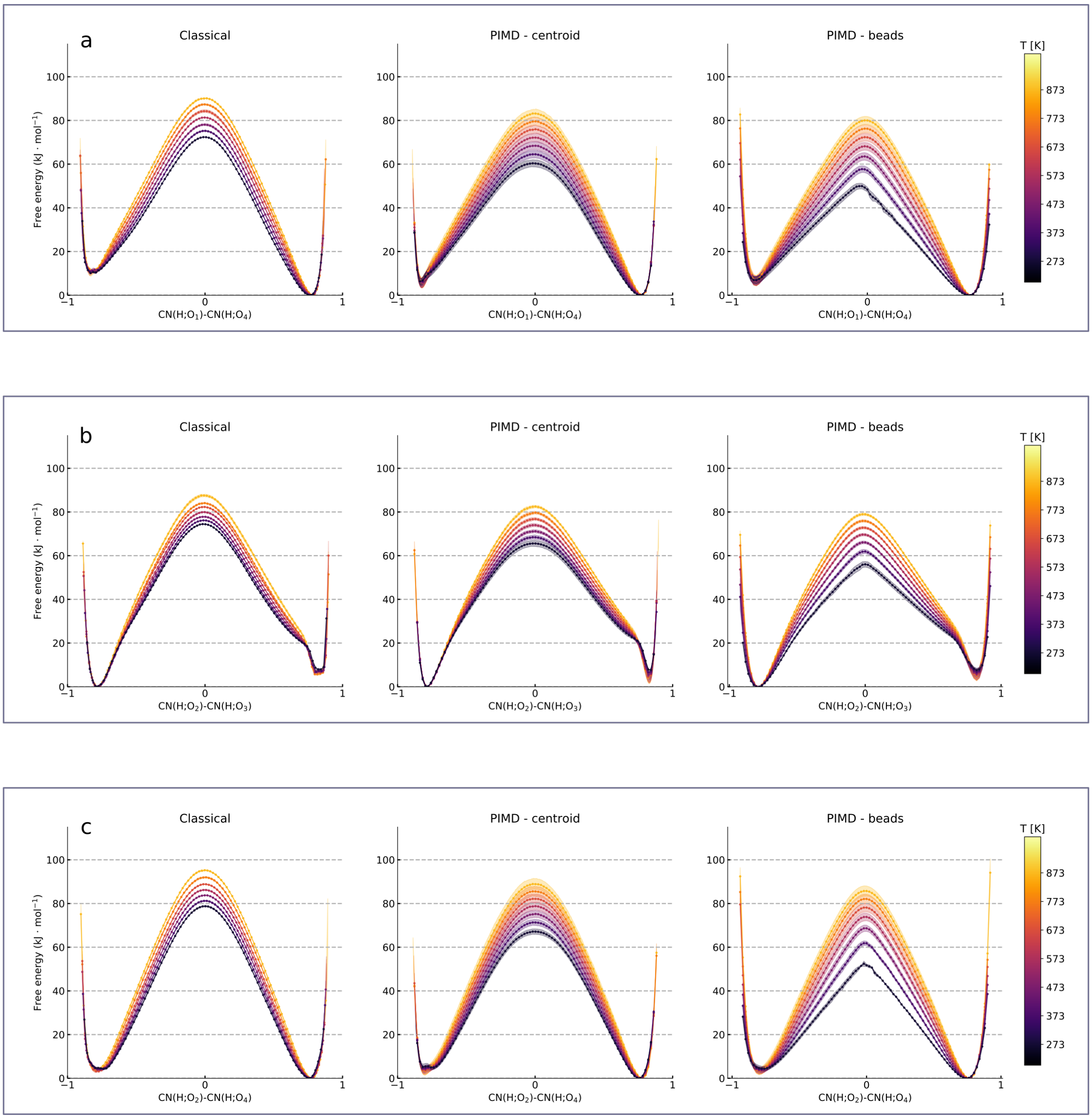

Supplementary Figure 15: Free energy profiles at temperatures ranging from 273 K to 873 K calculated classically and with NQEs as a function of the centroid collective variable and the bead collective variable. *a.* 1–4 hopping. *b.* 2–3 hopping. *c.* 2–4 hopping.

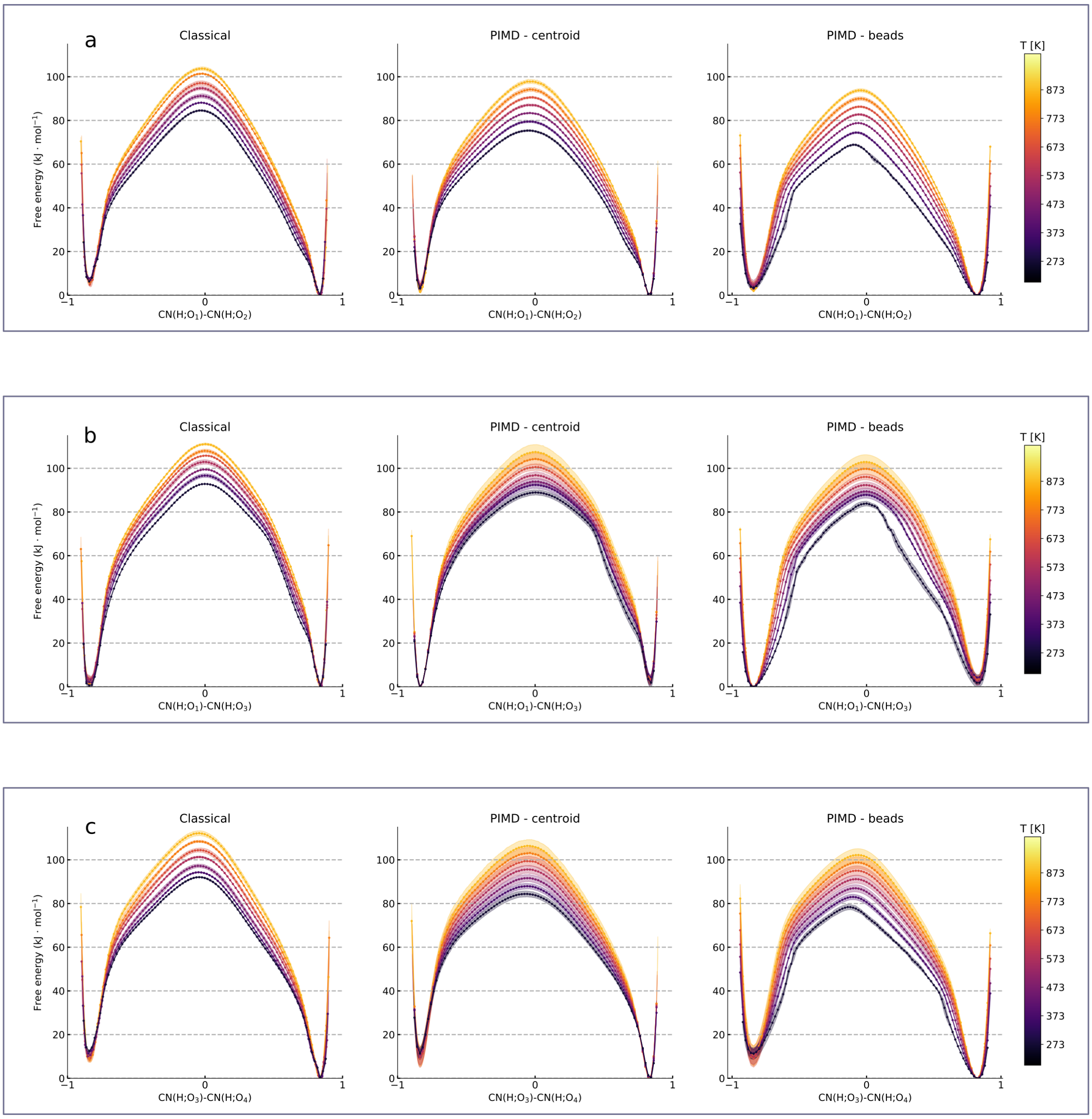

Supplementary Figure 16: Free energy profiles at temperatures ranging from 273 K to 873 K calculated classically and with NQEs as a function of the centroid collective variable and the bead collective variable. *a.* 1–2 hopping. *b.* 1–3 hopping. *c.* 3–4 hopping.

Supplementary Note 7.4 Quantum rate constants

Similarly to classical rate theory, the quantum rate can be obtained from the flux-side correlation function [52, 53]. However, as the sampling protocol of imaginary time PIMD only allows to compute static equilibrium properties and does not allow to compute time-dependent quantities, one needs to resort to approximate techniques such as ring polymer molecular dynamics (RPMD) to calculate quantum real-time correlation functions. In RPMD, the Hamiltonian of the classical isomorphism (Eq. S7.14) is used to approximately capture short-time quantum effects [54], which allows to formulate a ring polymer rate theory that resembles the classical rate theory in an extended phase space. Just as for the calculation of the free energy profiles (see Supplementary Note 7.3), the rate constant can be determined from the bead quantities or the centroid quantities, but now both formulations should yield the same result as the condition of having a particular bead in the product state or the centroid of the ring polymer is equivalent in the long-time limit [55]. The quantum rate constant within the RPMD approximation is thus given by

$$k_{\mathrm{BC}}(t) = \left\langle \dot{q}^{(c)}(0)\,\theta(q^{(c)}(t) - q^{(c)\ddagger})\right\rangle_{q^{(c)}(0)=q^{(c)\ddagger}} \frac{e^{-\beta F_{\mathrm{centroid}}(q^{(c)\ddagger})}}{\displaystyle\int_{-\infty}^{q^{(c)\ddagger}} \mathrm{d}q^{(c)}\, e^{-\beta F_{\mathrm{centroid}}(q^{(c)})}} \tag{S7.17}$$

$$= \frac{\displaystyle\sum_{k=1}^{P} \left\langle \dot{q}^{(k)}(0) \left[\frac{1}{P}\sum_{k'=1}^{P} \theta(q^{(k')}(t) - q^{(k')\ddagger})\right]\right\rangle_{q^{(k)}(0)=q^{(k)\ddagger}} e^{-\beta F_{\mathrm{quantum}}(q^{(k)\ddagger})}}{\displaystyle\sum_{k=1}^{P}\int_{-\infty}^{q^{(k)\ddagger}} \mathrm{d}q^{(k)}\, e^{-\beta F_{\mathrm{quantum}}(q^{(k)})}}, \tag{S7.18}$$

where the centroid formulation (Eq. S7.17) in particular shows an obvious resemblance with the classical rate formula (Eq. S3.6).

To test the equivalence between the two formulations, the quantum rate constant of the 2–3 hopping was calculated at 273 K using both of them. In the centroid formulation, 5,000 configurations with the centroid around the transition state ($q^* - 0.05 < q^{(c)}(0) < q^* + 0.05$) were extracted from an US simulation. Similarly to the classical rates (see Supplementary Note 6.2), a subsequent unbiased PIMD simulation was performed for 50 fs and the rate factor was calculated from Eq. S6.11. The full rate constant (Eq. S3.6) is then calculated using the centroid free energy profile at 273 K. For the bead formulation, 200,000 configurations with one bead at the transition state $q^* - 0.05 < q^{(i)}(0) < q^* + 0.05$ are extracted from an US simulation in which a single bead is constrained at the transition state. Once again, unbiased PIMD trajectories are then started from the configurations until the proton ends up in either the product or reactant basin. In the bead formulation, however, significantly more trajectories are required to obtain a converged rate. Since configurations in which one bead of the ring polymer is constrained at the transition state tend to orient all the remaining beads towards the product or reactant state (*i.e.* 'hang' over to one side of free energy barrier), the correlation between the velocity of the bead at the transition state and the chance of the bead or ring polymer ending up in the product state is much weaker than in the centroid formulation (see Supplementary Table 4). Therefore, many more trajectories are required to obtain adequate statistics and to converge the ensemble average in the rate factor. This phenomenon was also extensively reported by Manolopoulous et al. in Ref. [55].

Supplementary Table 4: Percentage of trajectories with an initial positive or negative collective variable velocity $\dot{q}(0)$ for the centroid or bead that end up in the product state $q(t_{\mathrm{end}}) > 0$ (with $t_{\mathrm{end}} = 50$ fs).

Formulation	$[\dot{q}(0) > 0 \mid q(t_{\mathrm{end}}) > 0]$	$[\dot{q}(0) < 0 \mid q(t_{\mathrm{end}}) > 0]$
Centroid	71.22	28.78
Bead	50.22	49.78

Due to the much smaller correlation between the initial collective variable velocity and the probability of ending up in the product state, the bead formulation yields a much lower rate factor. However, given that the free energy profile as function of the bead collective variable also exhibits a significantly lower barrier height, the balance between both effects results in a final rate constant that is approximately equal for

the bead and centroid formulation (see Supplementary Table 5). Yet, as the centroid formulation allows for a convergence of the rate factor with much less trajectories, this formulation is used for all the rate calculations reported in Supplementary Figure 17.

Supplementary Table 5: The rate factor (Eq. S6.11), free energy at the transition state $F(q^*)$ and the final rate constant k (Equation S3.6) for both the centroid and bead formulation for the 2–3 hopping at 273 K.

Formulation	Rate factor A [s^{-1}]	$F(q^*)$ [kJ·mol^{-1}]	Rate constant k_{BC} [s^{-1}]
Centroid	$4.26 \cdot 10^{12}$	65.57	22.82
Bead	$1.80 \cdot 10^{11}$	56.04	35.28

Supplementary Note 7.5 Quantum rate constants (deuterium)

Analogously to Supplementary Note 7.3 and Supplementary Note 7.4, the free energy profiles and rate constants were computed for deuterium hopping between each of the four oxygen atoms. Only the mass of the proton is changed, as the underlying potential energy surface (PES) governing the forces acting on the atoms remains unchanged. Remark that so far – to the best of our knowledge – this type of approach has been restricted to simple gas–phase reactions (sometimes adopting an MLP for the PES evaluations, see e.g. [56]). The bead profiles computed with deuterium (one proton, one neutron) at 273 K are compared with the protium (one proton, no neutrons) profiles in Supplementary Figure 18. The final deuterium rates were used to compute the equilibrium coverages (Supplementary Note 8), from which the final rate was obtained (see Figure 7 in the main text). The equilibrium coverages are graphically shown in Supplementary Figure 19.

Finally, to confirm the transferability of the accuracy of the MLP trained only on protium to the simulations performed with deuterium, snapshots were extracted from the US-MLP simulations of the 2–3 hopping at the highest temperature of 873 K. From these snapshots, the forces predicted by the MLP are compared with those recomputed at the reference DFT level of theory. The MAE on the forces as a function of the CV (CN(H;O_2)-CN(H;O_3)) is shown in Supplementary Figure 20. The errors are comparable with those from the previous validation on protium US-MLP simulations (see Supplementary Figure 14c), demonstrating the ability of the MLP (trained only on protium hopping) to accurately describe the hopping of deuterium between oxygen atoms.

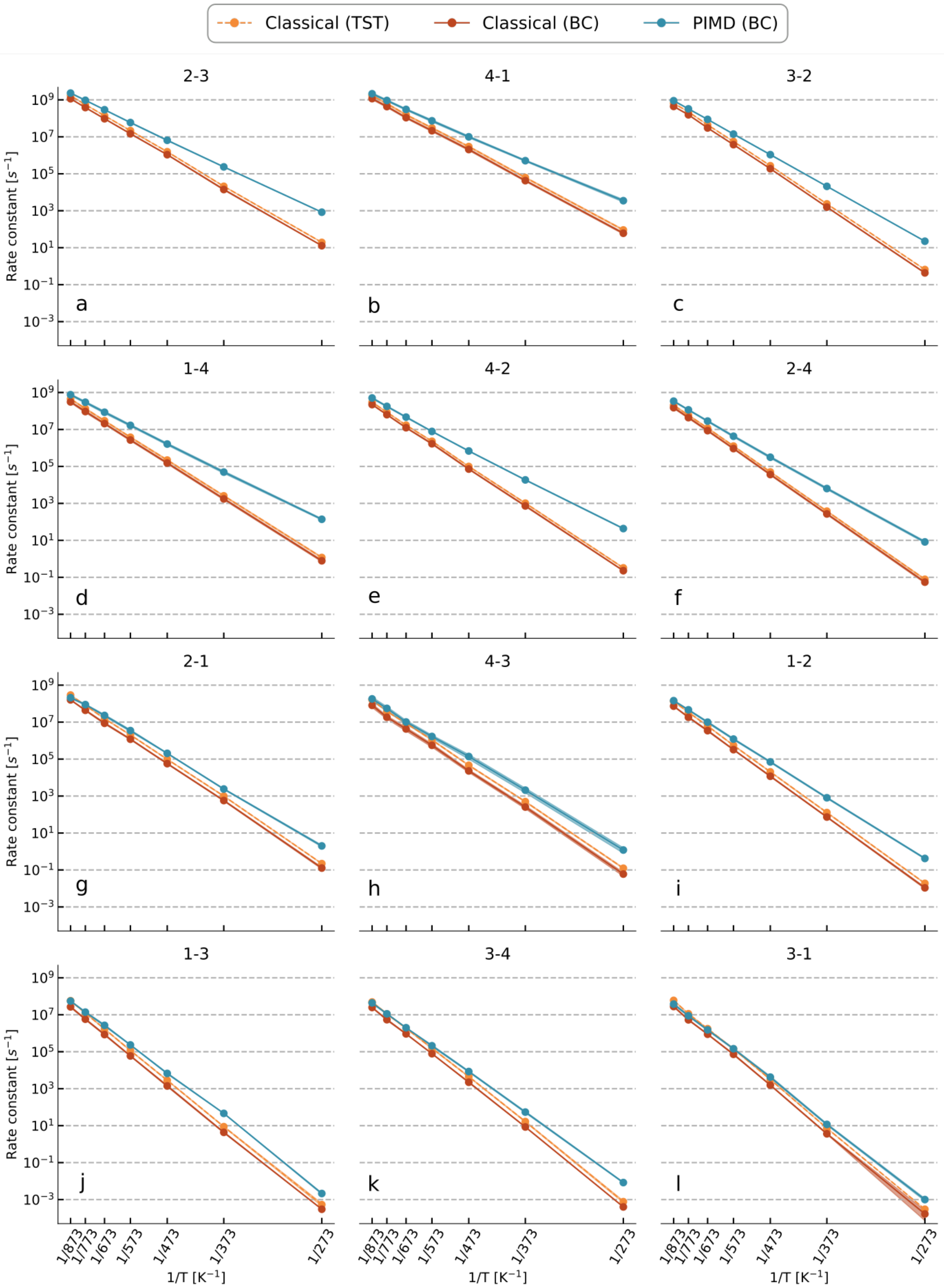

Supplementary Figure 17: Rate constants of all 6 hoppings in both directions shown in Arrhenius plots (log of the rate constant versus 1/T). Classical transition state theory rates are in orange, classical time-dependent rates are in red and time-dependent NQEs rates are in blue. *a.* 2–3 hopping. *b.* 4–1 hopping. *c* .3–2 hopping. *d.* 1–4 hopping. *e.* 4–2 hopping. *f.* 2–4 hopping. *g.* 2–1 hopping. *h.* 4–3 hopping. *i.* 1–2 hopping. *j.* 1–3 hopping. *k.* 3–4 hopping. *l.* 3–1 hopping. The hoppings are sorted from largest high temperature rate to lowest.

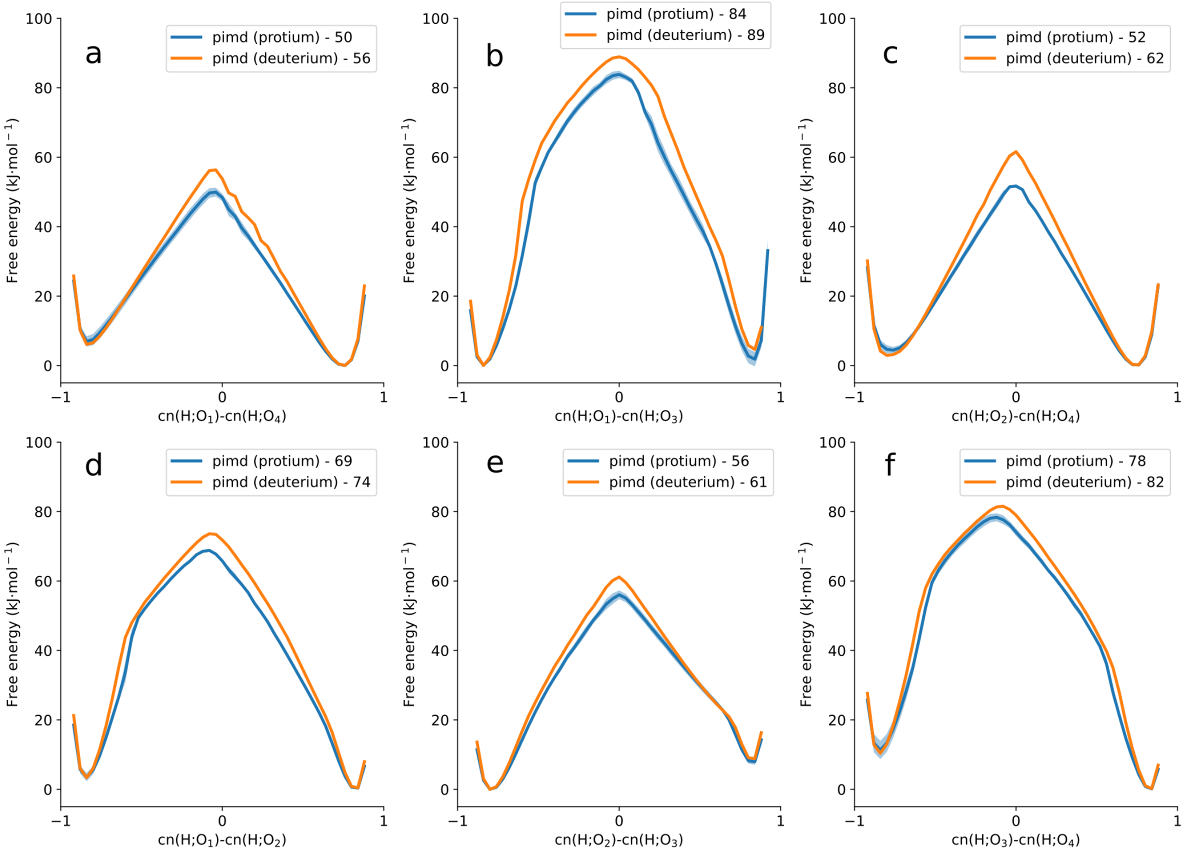

Supplementary Figure 18: Comparison between the bead free energy profiles of the protium (one proton, no neutrons) and the deuterium (one proton, one neutron) hoppings at a temperature of 273 K. *a.* 1–4 hopping. *b.* 1–3 hopping. *c.* 2–4 hopping. *d.* 1–2 hopping. *e.* 2–3 hopping. *f.* 3–4 hopping. The free energy at the transition state for each hopping is shown in the legend (kJ·mol^{-1}).

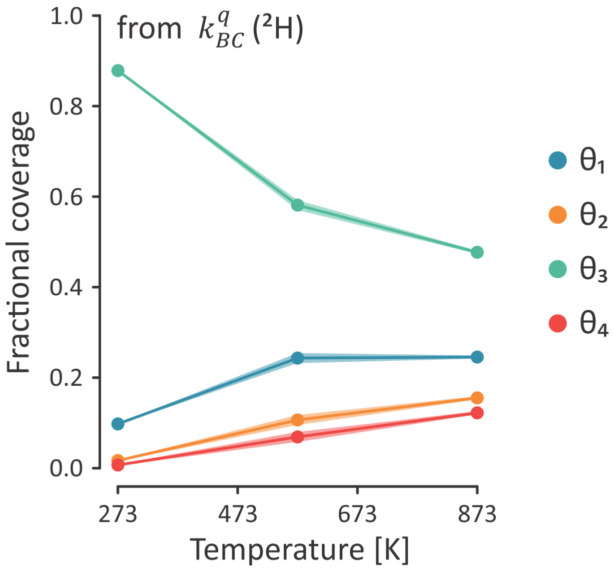

Supplementary Figure 19: Equilibrium coverages as function of temperature obtained from the PIMD kinetic constants ($k^q_{BC}(^2H)$) of the deuterium simulations.

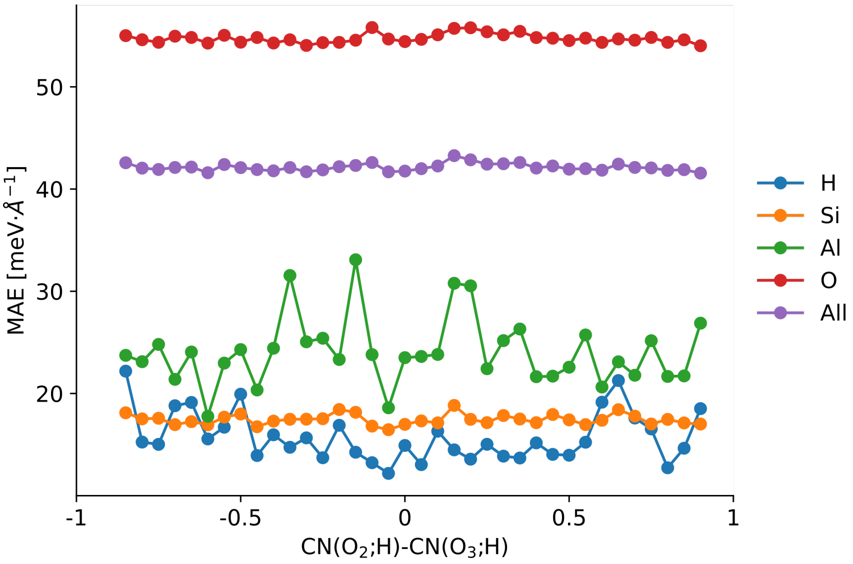

Supplementary Figure 20: The MAE on the forces predicted by the MLP on snapshots extracted from US-MLP simulations of the 2–3 hopping at 873 K as a function of the CV, compared to forces recomputed at the reference DFT level of theory. The errors are shown for all atoms in the system (All), as well as for each element (H, Si, Al, O).

Supplementary Note 8 Final hopping rate calculation

From the 12 kinetic constants characterizing the 6 forward and reverse hoppings between the 4 oxygen atoms in the first coordination sphere of the Al defect, it is possible to compute the equilibrium coverages θ_i of each site and, subsequently, the overall hopping rate:

$$r_{\text{hopping}} = \sum_{i=1}^{4} \sum_{j \neq i}^{4} k_{ij} \theta_i, \tag{S8.19}$$

which represents the overall number of hopping events per second. The rate of variation for the coverage of each site can be set equal to zero at equilibrium:

$$\frac{\mathrm{d}\theta_i}{\mathrm{d}t} = \sum_{j \neq i}^{4} (k_{ji}\theta_j - k_{ij}\theta_i) = 0. \tag{S8.20}$$

To normalize the site coverages, we can impose:

$$\sum_{i=1}^{4} \theta_i = 1, \tag{S8.21}$$

which immediately yields $\theta_4 = 1 - \sum_{n=1}^{3} \theta_i$. This leaves us with 4 equations, one for each oxygen site, in 3 unknowns (θ_1, θ_2 and θ_3). Because of the uncertainties on the kinetic constant, the system does not have a unique solution. The least squares solution can be found using the Moore-Penrose inverse coefficients matrix:

$$\mathbf{y} = K\boldsymbol{\theta} \tag{S8.22}$$

$$\boldsymbol{\theta} = (K^T K)^{-1} K^T \mathbf{y}, \tag{S8.23}$$

with

$$K = \begin{bmatrix} -(k_{12}+k_{13}+k_{14}+k_{41}) & k_{21}-k_{41} & k_{31}-k_{41} \\ k_{12}-k_{42} & -(k_{21}+k_{23}+k_{24}+k_{42}) & k_{32}-k_{42} \\ k_{13}-k_{43} & k_{23}-k_{43} & -(k_{31}+k_{32}+k_{34}+k_{43}) \\ k_{14}+k_{41}+k_{42}+k_{43} & k_{24}+k_{41}+k_{42}+k_{43} & k_{34}+k_{41}+k_{42}+k_{43} \end{bmatrix} \tag{S8.24}$$

$$\mathbf{y} = \begin{bmatrix} -k_{41} \\ -k_{42} \\ -k_{43} \\ k_{41}+k_{42}+k_{43} \end{bmatrix} \tag{S8.25}$$

$$\boldsymbol{\theta} = \begin{bmatrix} \theta_1 \\ \theta_2 \\ \theta_3 \end{bmatrix}. \tag{S8.26}$$

As a check, the least square equilibrium coverages were used to compute the residual $\mathrm{d}\theta_i/\mathrm{d}t$, for which we found values that are at least 5 orders of magnitude smaller than the final hopping rate $r_{hopping}$.

The uncertainties on the coverages were obtained by repeating the calculation 1000 times, where the employed k_i values are extracted from a normal distribution of $\log(k_i)$ with a standard deviation based on the 3 independent US runs. The results reported in Figure 6 of the main manuscript are the average of the 1000 repetitions with a 95% confidence interval given by twice their standard deviation. The uncertainty on the final rate was propagated from the uncertainties on the kinetic constants (where $\log(k_i)$ is assumed to be normally distributed) and on the equilibrium coverages.

The results for the various coverages as function of temperature are reported in Figure 6 of the main manuscript and are quite different from the results based on static calculations [27], which can be straightforwardly attributed to an improved description of temperature effects with dynamic techniques.

The differences in coverage between the classical and quantum rates might arise from a stronger interaction between the BAS and the zeolite oxygens in the 6-membered Si ring, when the BAS is located on O_3. To support this hypothesis, unbiased MLP (PI)MD simulations at 273 K were performed with the proton located on each of the 4 oxygen atoms neighboring the Al site for a total simulation time of 50 ps, with snapshots written out every 2 fs. Subsequently, 3 radial distribution functions (RDFs) were calculated from the obtained trajectories. The first RDF monitors the distance between the proton and the 6 oxygens bonded to the same Si and Al atoms as the oxygen atom on which the proton is located, thus $\mathbf{O}_3$Si–O(H)–Al$\mathbf{O}_3$ (denoted as H–O_{neigh}). The second RDF reports the pair distance distribution between the proton and all other oxygens in the zeolite unit cell, always excluding the oxygen atom on which the proton is located (denoted as H–O_{other}). The third RDF is simply the sum of the previous two, thus including all oxygen atoms in the unit cell except for the one where the BAS is located (denoted as H–O). For a proton located on each of the 4 oxygens surrounding the Al site, the RDFs are shown in Supplementary Figure 21. From the H–O_{other} RDF for oxygen 3, it follows that the proton can be additionally stabilized by hydrogen bonds with the framework oxygens on the opposite side of the 6-ring of the framework, at interatomic distances between 2 and 3 Å, while this is not the case for a proton located on the other oxygens. Including NQEs, the interatomic oxygen-proton distances are further reduced, possibly leading to an increased stabilization and, ultimately, to a larger coverage. This is also supported by the fact that, at higher temperatures, the coverage of O_3 with respect to O_1 becomes progressively closer to the classical case, in line with a reduced influence of NQEs.

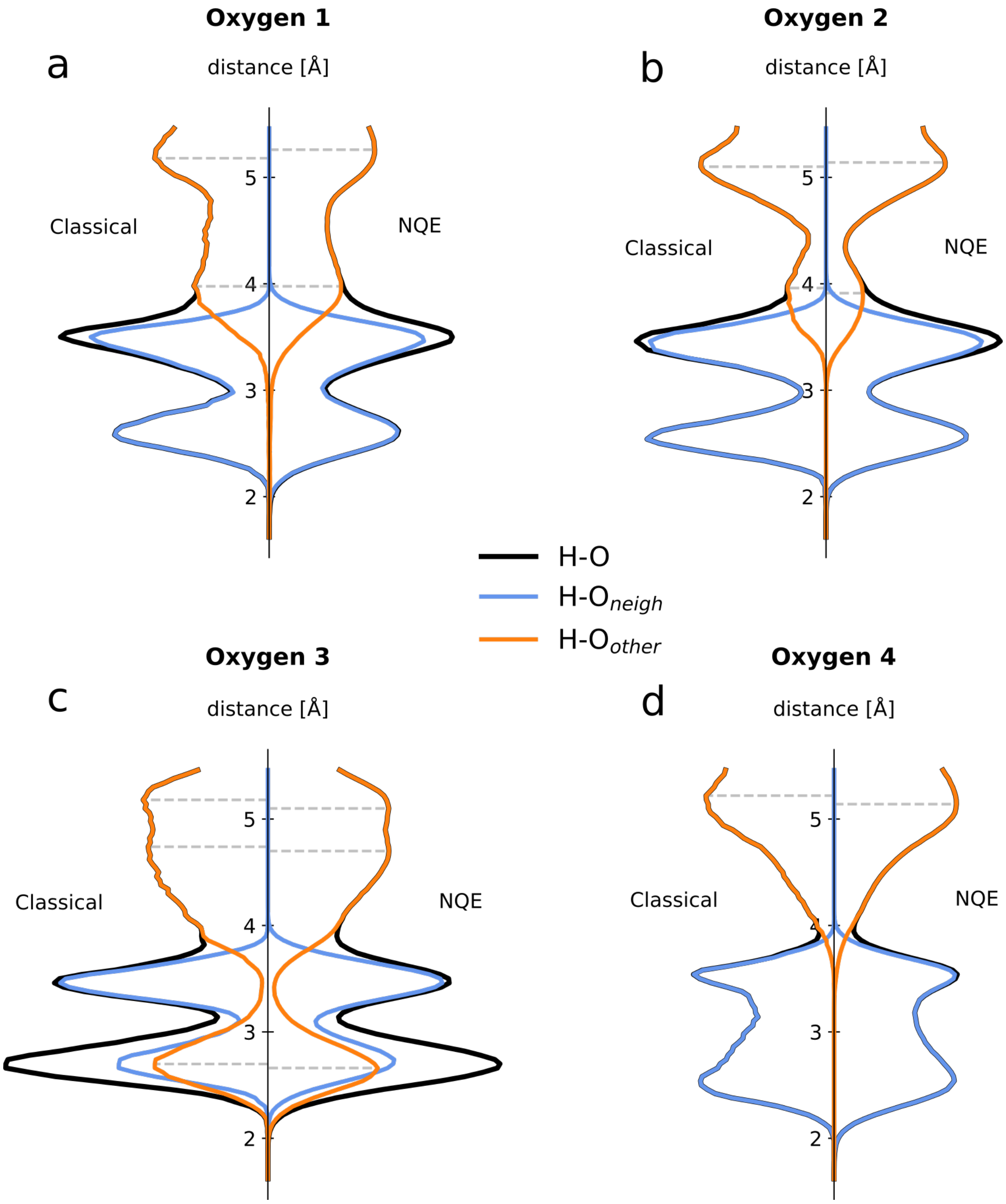

Supplementary Figure 21: O-H RDFs from classical and quantum MD simulations. The proton is located on oxygen atom 1 (*a*), 2 (*b*), 3 (*c*) or 4 (*d*). The RDFs are computed with all oxygen atoms in the zeolite (H–O, black), with the oxygens bonded to the same Si or Al atom as the oxygen atom on which the proton is located (H–O_{neigh}, blue) and with all oxygens excluding the ones included in H–O_{neigh} (H–O_{other}, orange).

Supplementary Note 9 MLP transferability to other zeolite topologies

To obtain an indication of the capability of the MLP to model other zeolite frameworks, the all-silica structures of CHA, AFX, FER, MFI and MOR were obtained from the IZA zeolite database [57]. The unit cell parameters of the different frameworks are reported in Supplementary Table 6. The choice for this set of zeolites was dictated by the wide usage of their topologies in catalysis research and industry [58], but also because the AFX topology represents a slight variation of the CHA topology while FER, MFI and MOR do not share any secondary building unit (SBU) with it. CHA was included as a validation structure and to assess if the MLP can deal with variations in the unit cell volume.

Supplementary Table 6: Unit cell parameters for the tested zeolite topologies as extracted from the IZA zeolite database [57]. These parameters were used for the NVT simulations to evaluate the MLP transferability.

Topology	a (Å)	b (Å)	c (Å)	α	β	γ
CHA	13.675	13.675	14.767	90.0	90.0	120.0
AFX	13.674	13.674	19.695	90.0	90.0	120.0
FER	19.018	14.303	7.541	90.0	90.0	90.0
MFI	20.090	19.738	13.142	90.0	90.0	90.0
MOR	18.256	20.534	7.542	90.0	90.0	90.0

For each structure, a 100 ps long NVT MD simulation at 873 K was performed both with the MLP and the DFT methodology. The unit cell parameters were kept the same as the crystallographic ones obtained from the IZA database (Supplementary Table 6). Because of the lack of hydrogen atoms, the time step was increased to 1 fs. The MLP did not show any identifiable nonphysical behavior, even for the frameworks that are very different from CHA. To quantitatively check the MLP performance, we monitored both the MAE between the MLP and the DFT forces and the differences in Si–Si and Si–O RDFs. A complete overview of the results is reported in Supplementary Figure 22.

Despite, not unsurprisingly, a worse performance for zeolites whose structure is very different from CHA, the MLP remains quite robust with a maximal error on the forces of 258 meV$\cdot$ Å^{-1} for MFI. The CHA framework is still excellently described by the MLP even if the cell volume is moderately different from the training one. Analogously, due to its similarity to CHA, the accuracy on AFX is also remarkable. Structurally, the MLP performs rather well, with all RDFs being almost perfectly superimposable, apart from small deviations for MFI. For MOR and FER the short c cell length does not allow to compute the RDF for distances above 3.5 Å, which is the region where the differences in the MFI RDFs become more noticeable.

Nonetheless, we find quite surprisingly that an MLP exclusively trained on CHA can maintain a reasonably physical behavior also for frameworks that are very different from the training one. This suggests that, with some additional training, it should also be possible to obtain a high accuracy for other zeolite topologies. Unfortunately, explicitly testing the proton hopping transferability would require additional expensive DFT US simulations and was therefore not further investigated.

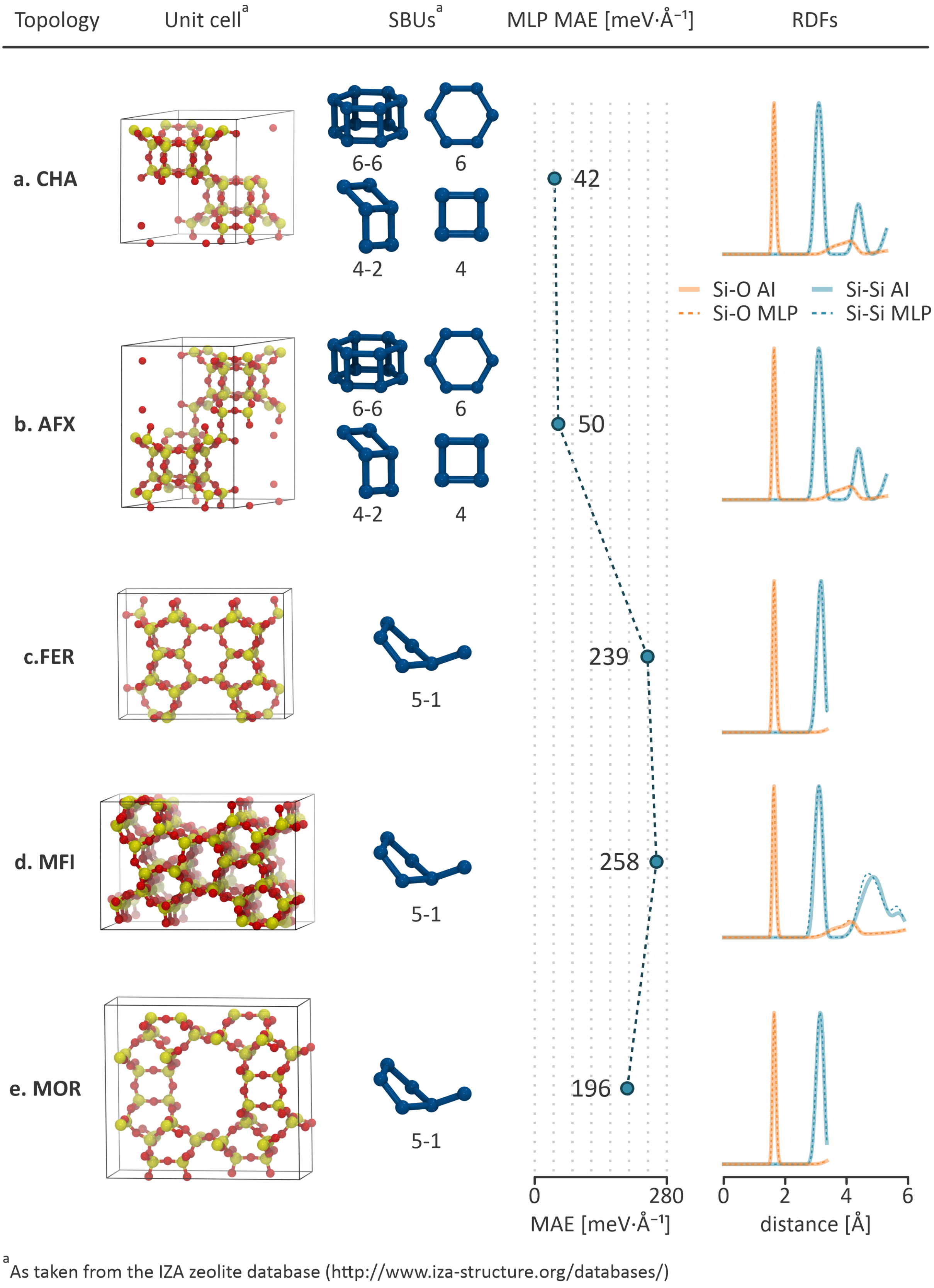

Supplementary Figure 22: Overview of the topologies investigated to assess the level of transferability of the MLP. For each topology (*a*–*e*), the unit cell is shown together with the SBUs that constitute the framework. The MAE on the forces was obtained by recomputing the forces of the DFT simulations with the MLP. The dotted line connecting the MAE values is to guide the reader's eye only. The Si–O and Si–Si normalized RDFs from the DFT and MLP MD simulations are shown in orange and blue, respectively. For FER and MOR, it is cut off at low distances because of the short c cell length (Supplementary Table 6).

Supplementary Note 10 MLP data efficiency

In order to validate the MLP FESs against the DFT ones at 873 K, well-converged simulations were required for the latter. Therefore, a simulation length of 50 ps was used for each umbrella and, subsequently, the MLP was trained on a data set based on the entire length of these trajectories. As showcasing the methodology was our primary interest, not much interest was devoted to data efficiency initially. However, it would be beneficial for future work to train accurate MLPs on substantially less DFT datapoints, consistently reducing the simulation time required for the training set generation. To investigate this possibility, we performed tests on the 2–3 hopping with the recently developed equivariant NequIP potential [59], which is highly data efficient. From the US DFT trajectories, data sets containing only the first 200 fs, 500 fs, 1 ps, 2 ps and 5 ps were extracted, sampled every 5 fs. A NequIP potential was trained on each of these data sets, using a cutoff radius of 5 Å, 4 interaction blocks, a maximum rotation order of 1, 32 features and a loss function combining the forces loss with a weight of 100 and an energy loss with a weight of 1. The trained models result in validation errors of 51, 46, 44, 43 and 41 meV$\cdot$ Å^{-1}, respectively. Subsequently, US simulations were performed with each MLP using the same umbrellas as in the DFT simulations. The resulting free energy profiles are shown on the right of Supplementary Figure 23 and compared with the DFT profile. Only the MLP trained on the first 200 fs deviates significantly from the DFT profile, while all others MLP FESs agree very well with the DFT one, where slight deviations are only visible around the product basin (CV $\approx$ 0.8). To understand the deviation of the MLP trained on only the first 200 fs of DFT results, a histogram of the collective variable of snapshots contained in the umbrella at the transition state is shown on the left of Supplementary Figure 23 for the different trajectory lengths. This figure demonstrates that for the 200 fs trajectory (blue histogram) gaps appear in the distribution of the CV. As no training data exists in these regions, the MLP inaccurately interpolates, resulting in a degraded performance. However, this also shows that as soon as the space of collective variables has been sampled without clear gaps (which is the case starting from a simulation length of 500 fs in this example), accurate MLPs can be derived.

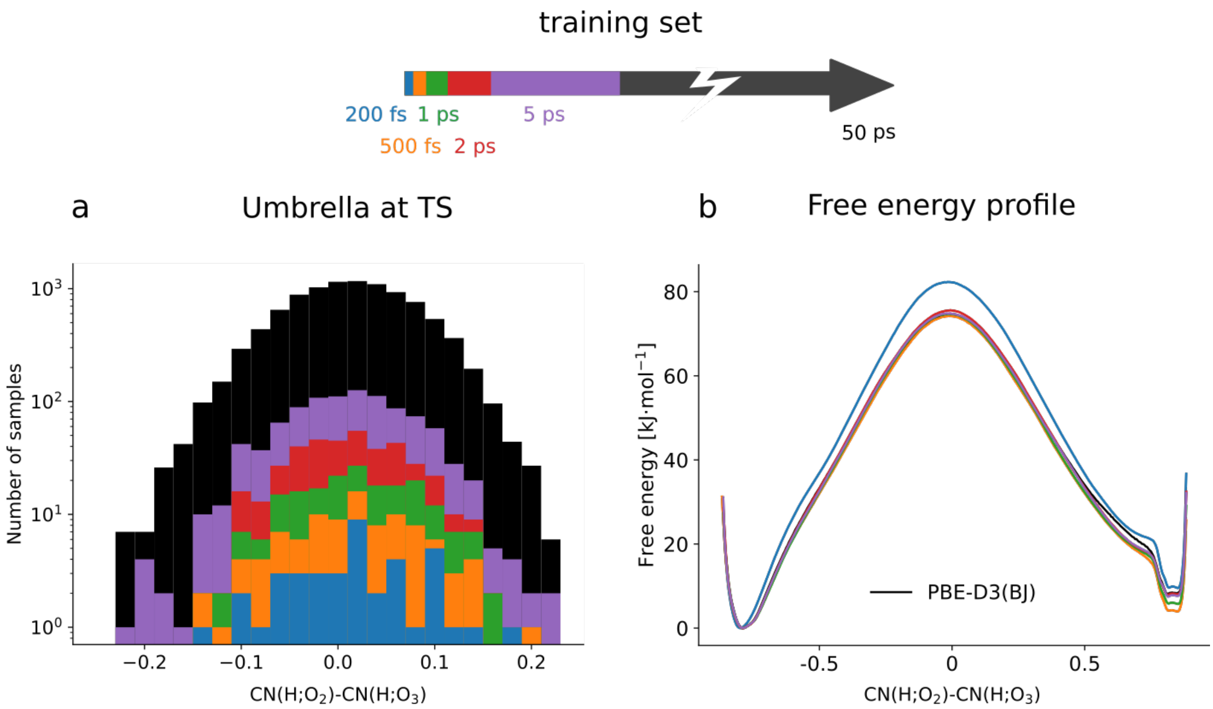

Supplementary Figure 23: Newer MLP architectures allow to drastically reduce the amount of training data. *a.* Histogram of the collective variable of snapshots from the DFT US simulations at the transition state from the first 200 fs, 500 fs, 1 ps, 2 ps, 5 ps and the full 50 ps of the trajectory. *b.* Free energy profiles calculated with different NequIP MLPs trained on the previously mentioned different trajectory lengths for umbrellas along the 2–3 hopping.

Supplementary References

1. Sierka, M. & Sauer, J. Finding transition structures in extended systems: A strategy based on a combined quantum mechanics–empirical valence bond approach. *The Journal of Chemical Physics* **112,** 6983–6996 (2000).
2. Sierka, M. & Sauer, J. Proton mobility in Chabazite, Faujasite, and ZSM-5 Zeolite catalysts. Comparison based on ab initio calculations. *The journal of physical chemistry B* **105,** 1603–1613 (2001).
3. Tuma, C. & Sauer, J. A hybrid MP2/planewave-DFT scheme for large chemical systems: proton jumps in zeolites. *Chemical Physics Letters* **387,** 388–394 (2004).
4. Osuga, R., Yokoi, T., Doitomi, K., Hirao, H. & Kondo, J. N. Infrared investigation of dynamic behavior of Brønsted acid sites on zeolites at high temperatures. *The Journal of Physical Chemistry C* **121,** 25411–25420 (2017).
5. Bucko, T., Gesvandtnerova, M. & Rocca, D. Ab initio calculations of free energy of activation at multiple electronic structure levels made affordable: An effective combination of perturbation theory and machine learning. *Journal of Chemical Theory and Computation* **16,** 6049–6060 (2020).
6. Baba, T., Inoue, Y., Shoji, H., Uematsu, T. & Ono, Y. Temperature-dependent lineshape of 1H magic-angle spinning nuclear magnetic resonance spectra of acidic hydroxyl groups in zeolites. *Microporous Materials* **3,** 647–655 (1995).
7. Sarv, P., Tuherm, T., Lippmaa, E., Keskinen, K. & Root, A. Mobility of the acidic proton in Broensted sites of HY, H-mordenite, and H-ZSM-5 zeolites, studied by high-temperature 1H MAS NMR. *The journal of physical chemistry* **99,** 13763–13768 (1995).
8. Baba, T., Komatsu, N., Ono, Y. & Sugisawa, H. Mobility of the acidic protons in H- ZSM-5 as studied by variable temperature 1H MAS NMR. *The journal of physical chemistry B* **102,** 804–808 (1998).
9. Ernst, H., Freude, D., Mildner, T. & Pfeifer, H. *High Temperature 1 H MAS NMR studies of the proton mobility in zeolites.* in *Proceedings of the 12th International Zeolite Conference* (1999), 2955–2962.
10. Franke, M. & Simon, U. Proton mobility in H-ZSM5 studied by impedance spectroscopy. *Solid State Ionics* **118,** 311–316 (1999).
11. Franke, M. & Simon, U. Characteristics of Proton Hopping in Zeolite H-ZSM5. *physica status solidi (b)* **218,** 287–290 (2000).
12. Ryder, J. A., Chakraborty, A. K. & Bell, A. T. Density functional theory study of proton mobility in zeolites: Proton migration and hydrogen exchange in ZSM-5. *The journal of physical chemistry B* **104,** 6998–7011 (2000).
13. Freude, D., Oehme, W., Schmiedel, H. & Staudte, B. NMR investigation of proton mobility in zeolites. *Journal of Catalysis* **32,** 137–143 (1974).
14. Fermann, J. T., Blanco, C. & Auerbach, S. Modeling proton mobility in acidic zeolite clusters. I. Convergence of transition state parameters from quantum chemistry. *The Journal of Chemical Physics* **112,** 6779–6786 (2000).
15. Kresse, G. & Hafner, J. Ab initio molecular-dynamics simulation of the liquid-metal–amorphous-semiconductor transition in germanium. *Physical Review B* **49,** 14251 (1994).
16. Kresse, G. & Furthmüller, J. Efficiency of ab-initio total energy calculations for metals and semiconductors using a plane-wave basis set. *Computational materials science* **6,** 15–50 (1996).
17. Kresse, G. & Furthmüller, J. Efficient iterative schemes for ab initio total-energy calculations using a plane-wave basis set. *Physical review B* **54,** 11169 (1996).
18. Blöchl, P. E. Projector augmented-wave method. *Physical review B* **50,** 17953 (1994).
19. Kresse, G. & Joubert, D. From ultrasoft pseudopotentials to the projector augmented-wave method. *Physical review b* **59,** 1758 (1999).
20. Perdew, J. P., Burke, K. & Ernzerhof, M. Generalized gradient approximation made simple. *Physical review letters* **77,** 3865 (1996).
21. Grimme, S., Antony, J., Ehrlich, S. & Krieg, H. A consistent and accurate ab initio parametrization of density functional dispersion correction (DFT-D) for the 94 elements H-Pu. *The Journal of chemical physics* **132,** 154104 (2010).

22. Heyden, A., Bell, A. T. & Keil, F. J. Efficient methods for finding transition states in chemical reactions: Comparison of improved dimer method and partitioned rational function optimization method. *The Journal of chemical physics* **123,** 224101 (2005).

23. Pulay, P. Convergence acceleration of iterative sequences. The case of SCF iteration. *Chemical Physics Letters* **73,** 393–398 (1980).

24. Ghysels, A., Van Neck, D. & Waroquier, M. Cartesian formulation of the mobile block Hessian approach to vibrational analysis in partially optimized systems. *The Journal of chemical physics* **127,** 164108 (2007).

25. De Moor, B. A. *et al.* Normal mode analysis in zeolites: toward an efficient calculation of adsorption entropies. *Journal of Chemical Theory and Computation* **7,** 1090–1101 (2011).

26. Ghysels, A., Verstraelen, T., Hemelsoet, K., Waroquier, M. & Van Speybroeck, V. TAMkin: A Versatile Package for Vibrational Analysis and Chemical Kinetics. *Journal of Chemical Information and Modeling* **50,** 1736–1750 (2010).

27. Kester, P. M., Crum, J. T., Li, S., Schneider, W. F. & Gounder, R. Effects of Brønsted acid site proximity in chabazite zeolites on OH infrared spectra and protolytic propane cracking kinetics. *Journal of Catalysis* **395,** 210–226 (2021).

28. Martyna, G. J., Tobias, D. J. & Klein, M. L. Constant pressure molecular dynamics algorithms. *The Journal of chemical physics* **101,** 4177–4189 (1994).

29. Woodcock, D. A. *et al.* Negative thermal expansion in the siliceous zeolites chabazite and ITQ-4: A neutron powder diffraction study. *Chemistry of materials* **11,** 2508–2514 (1999).

30. Martinez-Iñesta, M. M. & Lobo, R. F. Investigation of the negative thermal expansion mechanism of zeolite chabazite using the pair distribution function method. *The Journal of Physical Chemistry B* **109,** 9389–9396 (2005).

31. Bocus, M., Vanduyfhuys, L., De Proft, F., Weckhuysen, B. M. & Van Speybroeck, V. Mechanistic Characterization of Zeolite-Catalyzed Aromatic Electrophilic Substitution at Realistic Operating Conditions. *JACS Au* **2,** 502–514 (2022).

32. Vanduyfhuys, Louis. *ThermoLIB* version 1.0.0. `https://molmod.ugent.be/software/thermolib`.

33. Zhu, F. & Hummer, G. Convergence and error estimation in free energy calculations using the weighted histogram analysis method. *Journal of computational chemistry* **33,** 453–465 (2012).

34. Härdle, W. K. & Simar, L. *Applied multivariate statistical analysis* (Springer Nature, Berlin, Heidelberg, 2019).

35. Frenkel, D. & Smit, B. *Understanding molecular simulation: from algorithms to applications* (Elsevier, Amsterdam, 2001).

36. Peters, B. in *Reaction Rate Theory and Rare Events Simulations* 335–362 (Elsevier, Amsterdam, 2017). ISBN: 978-0-444-56349-1.

37. Bennett, C. H. in *Molecular dynamics and transition state theory: the simulation of infrequent events* 63–97 (American Chemical Society, 1977).

38. Chandler, D. Statistical mechanics of isomerization dynamics in liquids and the transition state approximation. *The Journal of Chemical Physics* **68,** 2959–2970 (1978).

39. Anderson, J. B. Statistical theories of chemical reactions. Distributions in the transition region. *The Journal of Chemical Physics* **58,** 4684–4692 (1973).

40. Bučko, T., Chibani, S., Paul, J.-F., Cantrel, L. & Badawi, M. Dissociative iodomethane adsorption on Ag-MOR and the formation of AgI clusters: an ab initio molecular dynamics study. *Physical Chemistry Chemical Physics* **19,** 27530–27543 (2017).

41. Bailleul, S. *et al.* Ab initio enhanced sampling kinetic study on MTO ethene methylation reaction. *Journal of Catalysis* **388,** 38–51 (2020).

42. Schütt, K. T., Sauceda, H. E., Kindermans, P.-J., Tkatchenko, A. & Müller, K.-R. SchNet – A deep learning architecture for molecules and materials. *The Journal of Chemical Physics* **148,** 241722 (2018).

43. Schütt, K. T. *et al.* SchNetPack: A Deep Learning Toolbox for Atomistic Systems. *Journal of Chemical Theory and Computation* **15,** 448–455 (2019).

44. Tribello, G. A., Bonomi, M., Branduardi, D., Camilloni, C. & Bussi, G. PLUMED 2: New feathers for an old bird. *Computer Physics Communications* **185,** 604–613 (2014).

45. Bonomi, M. *et al.* Promoting transparency and reproducibility in enhanced molecular simulations. *Nature methods* **16,** 670–673 (2019).

46. Kapil, V. *et al.* i-PI 2.0: A universal force engine for advanced molecular simulations. *Computer Physics Communications* **236,** 214–223 (2019).

47. Ceriotti, M., Parrinello, M., Markland, T. E. & Manolopoulos, D. E. Efficient stochastic thermostatting of path integral molecular dynamics. *The Journal of chemical physics* **133,** 124104 (2010).

48. Markland, T. E. & Ceriotti, M. Nuclear quantum effects enter the mainstream. *Nature Reviews Chemistry* **2,** 0109 (2018).

49. Laio, A. & Parrinello, M. Escaping free-energy minima. *Proceedings of the National Academy of Sciences* **99,** 12562–12566 (2002).

50. Laio, A. & Gervasio, F. L. Metadynamics: a method to simulate rare events and reconstruct the free energy in biophysics, chemistry and material science. *Reports on Progress in Physics* **71,** 126601 (2008).

51. Cendagorta, J. R., Shen, H., Bacić, Z. & Tuckerman, M. E. Enhanced Sampling Path Integral Methods Using Neural Network Potential Energy Surfaces with Application to Diffusion in Hydrogen Hydrates. *Advanced Theory and Simulations* **4,** 2000258 (2021).

52. Collepardo-Guevara, R., Craig, I. R. & Manolopoulos, D. E. Proton transfer in a polar solvent from ring polymer reaction rate theory. *The Journal of Chemical Physics* **128,** 144502 (2008).

53. Voth, G. A., Chandler, D. & Miller, W. H. Rigorous formulation of quantum transition state theory and its dynamical corrections. *The Journal of Chemical Physics* **91,** 7749–7760 (1989).

54. Craig, I. R. & Manolopoulos, D. E. Quantum statistics and classical mechanics: Real time correlation functions from ring polymer molecular dynamics. *The Journal of Chemical Physics* **121,** 3368–3373 (2004).

55. Craig, I. R. & Manolopoulos, D. E. A refined ring polymer molecular dynamics theory of chemical reaction rates. *The Journal of Chemical Physics* **123,** 034102 (2005).

56. Liu, Y. & Li, J. An accurate potential energy surface and ring polymer molecular dynamics study of the Cl + CH_4 → HCl+ CH_3 reaction. *Physical Chemistry Chemical Physics* **22,** 344–353 (2020).

57. *IZA database of zeolite structures* http://www.iza-structure.org/databases/. Accessed: 2022-03-15.

58. Van Speybroeck, V. *et al.* Advances in theory and their application within the field of zeolite chemistry. *Chemical Society Reviews* **44,** 7044–7111 (2015).

59. Batzner, S. *et al.* E (3)-equivariant graph neural networks for data-efficient and accurate interatomic potentials. *Nature communications* **13,** 1–11 (2022).
